# The place of millet in food globalization during Late Prehistory as evidenced by new bioarchaeological data from the Caucasus

**DOI:** 10.1038/s41598-021-92392-9

**Published:** 2021-06-23

**Authors:** Lucie Martin, Erwan Messager, Giorgi Bedianashvili, Nana Rusishvili, Elena Lebedeva, Catherine Longford, Roman Hovsepyan, Liana Bitadze, Marine Chkadua, Nikoloz Vanishvili, Françoise Le Mort, Kakha Kakhiani, Mikheil Abramishvili, Giorgi Gogochuri, Bidzina Murvanidze, Gela Giunashvili, Vakhtang Licheli, Aurélie Salavert, Guy Andre, Estelle Herrscher

**Affiliations:** 1 Laboratory of Prehistoric Archaeology and Anthropology, University of Geneva, Carl-Vogt 66, 1211 Geneva 4, Switzerland; 2 EDYTEM, UMR 5204, University of Savoie Mont-Blanc, Pôle Montagne, 73376 Le Bourget du Lac, France; 3 Georgian National Museum, 14 Uznadze Str., 0102 Tbilisi, Georgia; 4 Laboratory of Nature Sciences in Archaeology, Institute of Archaeology of Russian Academy of Sciences, Dm. Ulyanova, 19, Moscow, 117036 Russia; 5 Department of Archaeology, University of Sheffield, Minalloy House, 10 - 16 Regent Street, Sheffield, S1 3NJ UK; 6 Institute for Archaeology and Ethnography, National Academy of Sciences of the Republic of Armenia, Charents Str. 15, 0025 Yerevan, Armenia; 7 Institute of History and Ethnology, Anthropological Research Laboratory, Tbilisi State University, Tbilisi, Georgia; 8 University of Lyon, Archéorient (UMR 5133 CNRS/ University of Lyon 2), Maison de l’Orient et de la Méditerranée-Jean Pouilloux, Lyon, France; 9 Institute of Archaeology, Ivane Javakhishvili Tbilisi State University, Tbilisi, Georgia; 10 AASPE, UMR 7209, Muséum National D’Histoire Naturelle, 55 rue Buffon, CP56 Paris, France; 11 Aix Marseille Univ, CNRS, Minist Culture, LAMPEA, UMR 7269, MMSH CS 90412, 5 rue du Château de l’Horloge, 13097 Aix-en-Provence, Cedex 2, France

**Keywords:** Biochemistry, Archaeology, Plant domestication

## Abstract

Two millets, *Panicum miliaceum* and *Setaria italica*, were domesticated in northern China, around 6000 BC. Although its oldest evidence is in Asia, possible independent domestication of these species in the Caucasus has often been proposed. To verify this hypothesis, a multiproxy research program (Orimil) was designed to detect the first evidence of millet in this region. It included a critical review of the occurrence of archaeological millet in the Caucasus, up to Antiquity; isotopic analyses of human and animal bones and charred grains; and radiocarbon dating of millet grains from archaeological contexts dated from the Early Bronze Age (3500–2500 BC) to the 1^st^ Century BC. The results show that these two cereals were cultivated during the Middle Bronze Age (MBA), around 2000–1800 BC, especially *Setaria italica* which is the most ancient millet found in Georgia. Isotopic analyses also show a significant enrichment in ^13^C in human and animal tissues, indicating an increasing C_4_ plants consumption at the same period. More broadly, our results assert that millet was not present in the Caucasus in the Neolithic period. Its arrival in the region, based on existing data in Eurasia, was from the south, without excluding a possible local domestication of *Setaria italica*.

## Introduction

Today, there are many genetic, archaeobotanical or biochemical resources to track the domestication processes and diffusion of plants and animals. Among them, the millets, *Panicum miliaceum* and *Setaria italica,* are two species that have their origins in China, but their path to Europe has been the subject of numerous studies for several years. Across Asia, researchers have looked at millets to describe the dispersal of peoples and languages^1^, to address the concept of food globalization^2^, to understand how these cereals have adapted to different environments^3,4^ and to investigate the sedentary, semi-nomadic, or nomadic lifestyles of the early agropastoral communities^5,6^. This article focuses on the Caucasus, a region that has hitherto not yet been explored in this respect, and that requires fresher data to understand the process of millet dispersal. Millets have long been considered present in the European Neolithic, and also in the Caucasus. In Europe, new radiocarbon dates made directly on millet grains and human skeletal stable isotope analyses show that finally, these two small cereals arrive during the Bronze Age^7–9^. In the light of these recent studies, a multi-proxy project delivers new dates, which specify the period of appearance of millet in the Caucasus and redefine the modalities of its diffusion from East Asia. These dates are corroborated by isotopic data, which prove to be a strong indicator of the consumption of millet, a C_4_ plant. Finally, archaeobotany, radiocarbon dates and isotopic data also reveal that one of the species, *Setaria italica*, could have been domesticated locally in the Western Caucasus.

In the following, “millet” designates two species, *Panicum miliaceum* (common millet) and *Setaria italica* (foxtail millet). The terms millets, small millets, *Panicum* or *Setaria* only refer to these two cultivated species.

Despite the current minor importance of the crops, small millets are nutritionally superior to large-grained cereals like wheat or barley, in terms of proteins, minerals, and vitamins. The grains can be stored for a long time; they have good productive returns and need little management. *Panicum* and *Setaria* are two spring warm-weather crops and rainfed summer crops, which do not need irrigation. Two of the main properties of millet cultivation, which probably heightened its attractiveness to prehistoric populations, are their short growing period, between 40 and 90 days, and tolerance to aridity. Millets require about half the water compared to wheat, and their cultivation does not require plowing due to its shallow roots. Thus, millets are very suitable for cultivation by semi-nomadic societies both in the past and still today, as they are low-investment agricultural crops. Numerous studies have been carried out on chronology and routes of the spread of millet from China to Europe, especially across the central Eurasian steppe regions and mountain corridors^3,10–12^. The oldest cultivated grains of *Panicum* and *Setaria* were found at several sites in North China between 6000 and 5500 cal BC^1,13,14^. Millet domestication probably took millennia and the manner of its domestication is still subject to debate^14–16^. For example, phytoliths and biomolecular components of millet were also found on the Neolithic site of Cishan (Hebei province, Northern China) between ca. 8300 and ca. 6700 cal BC^17^. Starch and phytoliths are not species specific, and several commentators highlight poorly defined contexts which seem insufficient to confirm this early domestication^3,15,18^. Between 3500 and 2200 BC, millet agriculture is attested in the high-altitude environment of the Tibetan Plateau, which demonstrates that millets are able to adapt to different ecological zones. *Setaria* and *Panicum* provide the earliest evidence for agricultural products in this region, before the introduction of wheat, barley and pea^4^.

**Figure 1:**
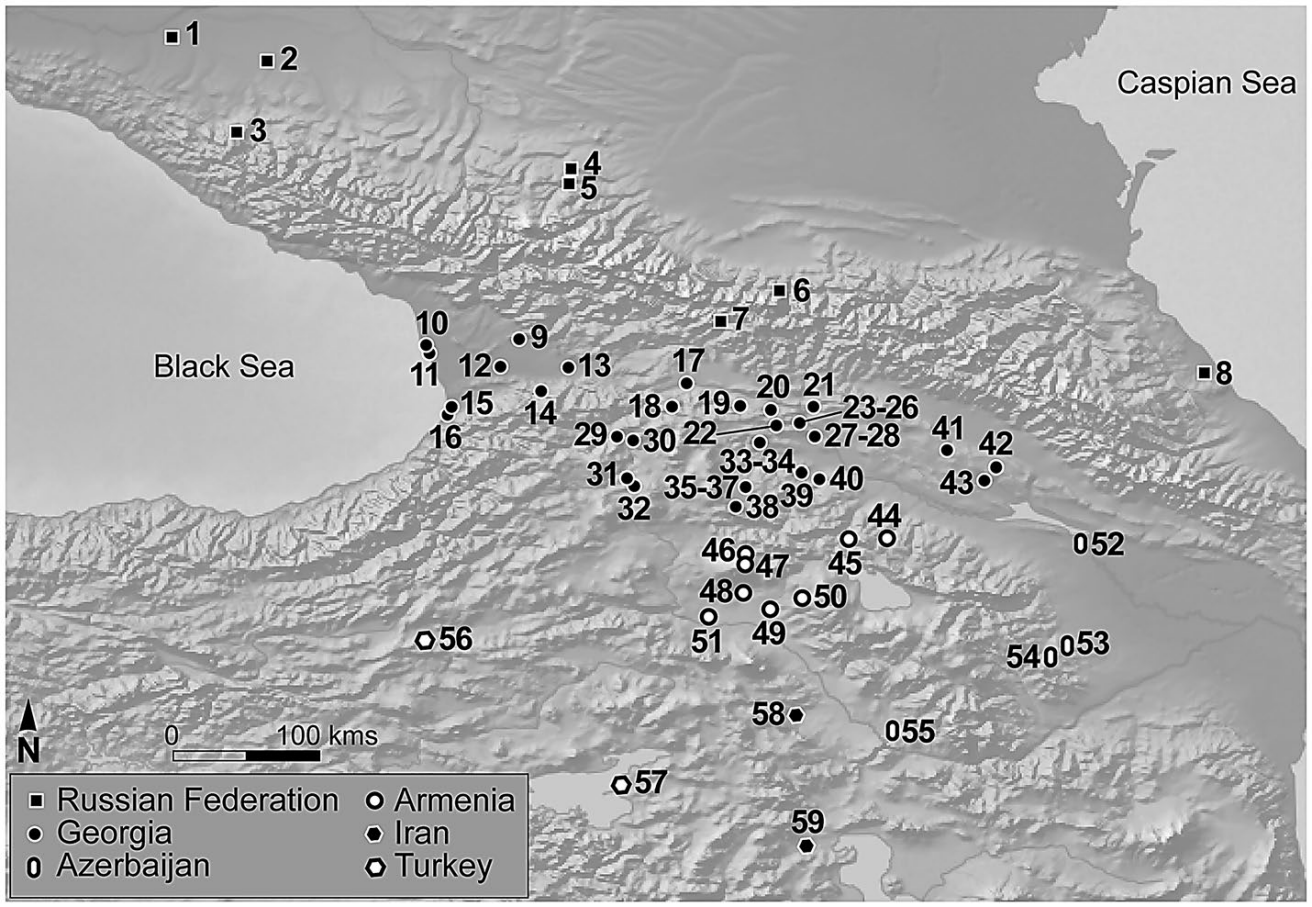
Map of the Caucasus showing all the sites with millet and/or where isotopic analysis were performed on humans and/or animal bones. **1.**Chishkho; **2.**Lesnoe; **3.**Guamsky Grot; **4.** Kislovodsk; **5.**Kabardinka-2; **6.**Koban; **7.**Chidgom; **8.**Velikent I; **9.**Etseri village; **10.**Dikha-Gudzuba; **11.**Pichori; **12.**Nosiri; Megrelia; **13.**Gabashvilis Gora; **14.**Vani; **15.**Choloki; **16.**Namcheduri; **17.**Tsagvli; **18.**Natsargora; **19.**Gudabertka; **20.**Grakliani Gora; **21.**Abanoskhevi; **22.**Tsikhia Gora; **23.**Tsitsamuri; **24.**Natakhtari; **25.**Samtavro; **26.**Narekvavi; **27.**Treli/Treligorebi; **28.**Digomi; **29.**Atskuri; **30.**Tiselis Seri; **31.**Akhchia; **32.**Bertkama-Chalis kurgan; **33.**Chala; **34.**Kobala; **35.**Gantiadi; **36.**Dalari; **37.**Karataki; **38.**Irganchai, Tashiri; **39.**Aruchlo 1; **40.**Imiris-Gora; **41.**Khramebi; **42.**Noname-Gora; **43.**Ciskaraant Gora; **44.**Sev-Sev Kareri Blur; **45.**Yenokavan-2; **46.**Gegharot; **47.**Tsaghkahovit; **48.**Nerkin Naver; **49.**Karmir Blur; **50.**Aramus; **51.**Argishtikhinili; **52.**Mingechevir; **53.**Uzerlik-Tepe; **54.**Khojaly; **55.**Kültepe 1; **56.**Sos Höyük; **57.**Ayanis; **58.**Haftavan Tepe (Background map made with Natural Earth @ naturalearthdata.com). References and details in SI appendix tab. S1.

While it is now established that the most ancient occurrences of *Panicum* and *Setaria* come from East Asia, other domestication centers for millets have been mentioned, including the Caucasus^19–23^. This region, situated between the Black and Caspian Seas and intersected by the Greater Caucasus Mountains has been presented as either a land bridge or a barrier between the Eurasian steppes and Western Asia (Fig. 1). The first farmers settled in the Caucasus at the beginning of the sixth millennium BC (*Aratashen-Shulaveri-Shomutepe* Neolithic culture), some 3500 years after the earliest manifestations of agriculture in south-eastern Anatolia. They cultivated a broad crop spectrum: cereals such as naked and hulled barley (*Hordeum vulgare*), emmer (*Triticum turgidum* subsp. *dicoccon*), free-threshing wheat (*T. aestivum/durum*) and einkorn (*Triticum monococcum*), as well as several pulses. Archaeological finds of sickles and sandstone grinders attest to widespread agricultural activities and processing of cereals across the Caucasus^24–27^. Present research shows that glume wheats and barley were introduced into the Caucasus from the Middle-East^28^. The Caucasus however, have historically been suggested as a center for crop domestication^20,22^. In particular, concerning the early dominance of naked wheat, rare at Neolithic sites in Western Asia, genetic analyses of modern bread wheat and distribution of wild *Aegilops tauschii* all indicate that hexaploid wheat originated in the Caucasus^22,23,29^. Whether millets were also locally domesticated in the Caucasus has been recently questioned^30^.

A multidisciplinary project, combining several approaches based on archaeobotanical studies, radiocarbon dates and isotopic analyses, was established to provide new data on the origin and development of millet cultivation in the Caucasus between the Neolithic and the Iron Age. The objective was to track this cereal at Caucasian archaeological sites, in order to evaluate the role of the Caucasus in the spread of millet and to propose new hypotheses on the manner of its diffusion from Central Asia to Europe.

## Results and discussion

### Evidences of millet plant remains in the Caucasus: A critical review

Several publications report the presence of millet grains (not always well identified) in the Caucasus since the Neolithic, before 5000 BC^19–22,30–32^. Older publications, notably that of G. N. Lisitsina (1984) propose a domestication of common millet in the Caucasus (more widely in the Soviet Union). In this context, a discussion on the appearance and spread of millet in the Caucasus requires a critical assessment of this evidence and a new set of archaeobotanical data. In this paper, our inventory of millet remains includes grains of *Panicum miliaceum* and *Setaria italica* derived from published literature and new discoveries from recent and older unpublished excavations. This represents 40 occurrences of millets, distributed in the Russian Federation, Georgia, Armenia, Azerbaijan, Turkey and Iran. The chronology of these remains extends between the fifth millennium BC to first century AD (SI appendix tab. S1). Many early published accounts of millets provide unreliable evidence because the dates are not precise, catalogues do not give quantitative evaluation and no verification is possible due to the difficulty of recovering the material. As an example, in earlier publications, millet is listed as present at Aruchlo-1, an important Neolithic site in Georgia, dated to 6050–5200 cal BC^20,30^, however according to the archaeobotanists working on this site, the grains are actually wild *Setaria* (R. Neef and N. Rusishvili^34^). At Dikha-Gudzuba in Georgia, mentioned in Lisitsina and Prishchepenko (1977), *Panicum* grains are supposed to have been identified, but this cereal is absent according to N. Rusishvili, who completed the archaeobotanical analysis of the site^34^. This critical review is strongly supported by current analyses: beside wheat and barley, Neolithic sites recently excavated revealed pulses (lentil, grass pea and bitter vetch) and flax but none of them uncovered any millet, except wild *Setaria*^25,26,34,35^. Millets are also absent in archaeobotanical spectrum from recently excavated Kura-Araxes sites dated from Early Bronze Age^36,37^.

### First occurrence of millet in the Caucasus: new radiocarbon and isotopic data

#### Archaeobotanical material directly radiocarbon dated

**Figure 2:**
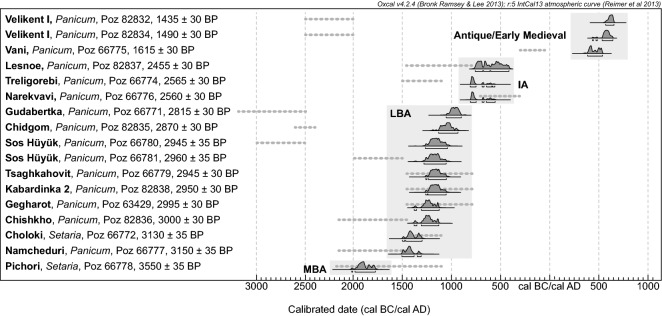
Calibrated radiocarbon dates made directly on millet grains. MBA: Middle Bronze Age; LBA: Late Bronze Age; IA: Iron Age. Chronological landmarks from Sagona 2016^27^. The grey dotted lines indicate the expected time period of the levels from which the millet samples are taken, according to archaeological data. Dated are calibrated using OxCal 4.2.4 software using IntCal13 atmospheric curve (references and details in SI appendix fig. S1 and tab. S2; dataset S1).

17 samples of millet grains (15 *Panicum* and 2 *Setaria*) were selected from 15 archaeological sites in Georgia, Armenia, Russian Federation and North-East Turkey (Fig. 1, 2). All the remains are charred and come from domestic contexts (occupation layers, burned house, fireplaces), generally dated by the archaeological material and radiocarbon, to between the Neolithic and Iron Age (SI appendix fig. S1, tab. S2; SI dataset S1). The 17 radiocarbon dates performed on millet give a chronological range between 3350 ± 35 BP to 1435 ± 30 BP. Ten samples offer more recent dating than expected by the archaeological context. For instance, millet from the Iron Age site of Vani (Western Georgia) instead dates to Late Antiquity, around 400 cal AD. This is a surprising result because according to the archaeological data, the city was destroyed in the mid-first century BC^38^. In the Early Bronze Age occupation layers of Velikent (Dagestan), millet is particularly time-shifted, with two intrusive samples from the Middle Ages. Moreover, at Velikent, isotope analyses do not indicate the consumption of C_4_ plants during the Bronze Age^39^. *Panicum* from Sos Höyük, in North-eastern Turkey does not date to the Early and Middle Bronze Ages as expected, but to the Late Bronze Age^40^.

Seven dates are in conformity with the occupation from which they were sampled. Located on the Black Sea coast, Pichori provides the most ancient dates for millet cultivation in the Caucasus with *Setaria italica* dated to between 2011 and 1771 cal BC. Over one hundred *Setaria* grains found with barley in a domestic context^34^, as well as the grain morphology (SI appendix fig. S2), leave no doubt as to the cultivated form of this species. At Namcheduri and Choloki, two sites located further south on the coast, grains of common millet and foxtail millet are dated to between 1500 and 1300 cal BC, that is the beginning of the Late Bronze Age (LBA). Several sites widely distributed throughout the Caucasus provided millet samples dated from the LBA in a range between 1400 and 900 cal BC, followed by some Iron Age millet dates (800–400 cal BC), and finally more recent intrusions in Vani and Velikent, from Late Antiquity and the very beginning of the Middle Ages (Fig. 2). In the Caucasus, several additional direct dates on millet grains should be mentioned (SI appendix tab. S2): at Grakliani Gora near Tbilisi, *Panicum* is dated to 1108–896 cal BC (V. Licheli and P. Biagi); in Guamsky Grot, situated in the northwestern Caucasus, *Panicum* grains were dated to 1110–908 cal BC^41^; and in Noname Gora, in Eastern Georgia, millet has been dated to the Iron Age 800–400 cal BC (R. Neef). In addition to our data, these 14C dates confirm *Panicum* cultivation in the Caucasus during the LBA.

Outside of the Caucasus, several presumed Neolithic/Bronze Age *Panicum* grains were also directly dated. In Ukraine, a recently dated grain of millet from the Neolithic site of Kamyane Zavallia has been dated to 264–541 cal AD (GifA19189a-1635 ± 45 BP^42^]. Furthermore, in Usatovo, a site dated to 3500–3000 BC on the Black Sea coast in Ukraine, a re-examination of presumed impressions of millet in clay using SEM techniques and experimentation showed that the marks may not have been made by a cereal imprint^43^. Numerous broomcorn millet grains from Central and Eastern European Neolithic structures were recently directly radiocarbon dated. None of these grains returned Neolithic dates; the oldest remains date back to the Middle Bronze Age (MBA) and LBA, during the second half of the 2nd millennium^7,8^. In the Caucasus, as described above, all the charred millet grain directly dated as part of our project, which were presumed to be EBA (Kura-Araxes Culture) or MBA I (2500–2000 BC), are younger. Our results confirm that archaeological plant material, especially small botanical remains, can be intrusive and that a direct dating of macroremains are needed to determine their chronology.

#### Isotopic analyses

Unlike the other crops grown in the same regions, *Panicum* and *Setaria* are both C_4_ plants that utilize the Hatch-Slack photosynthetic pathway which results in higher δ^13^C values in the plant tissue than C_3_ plants. Consequently, it results in measurable differences in the bone collagen of consumers, human and animal, of both these cereals^44^. Diets based on the consumption of C_4_ plants are easily identifiable by higher carbon isotope ratios^9,45^. In the Caucasus, wild C_4_ plants grow in several ecosystems, mainly located in the more arid parts of the region^46^. If these wild C_4_ plants were consumed by domestic animals, they could have contributed to higher bone collagen carbon isotopes ratios. Nevertheless, all the selected sites for which isotopic analysis have been carried out, fall in temperate vegetation belts in which C_4_ plants are minor components (see vegetation composition in^48,49^), except one site (Khramebi, located in Kakheti, where C_4_ plants occur in the surrounding steppes). In order to track the characteristics of the cereals consumed (wheat/barley *vs*. millet), stable isotope analyses (δ^13^C, δ^15^N) were carried out on samples collected in Georgia and dated from the Middle Bronze Age to the Late Iron Age.

**Figure 3:**
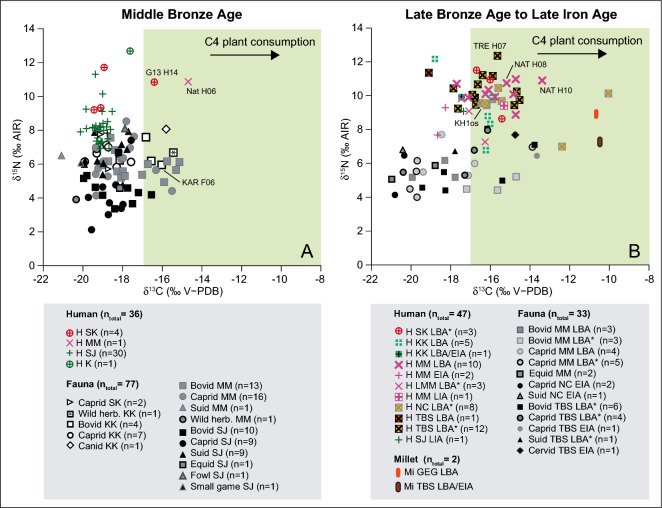
Animal and human stable carbon and nitrogen isotope ratios according to geographical area for Middle Bronze Age (**A**) and Late Bronze Age to late Iron Age (**B**). H: Human, Mi: millet; Geographical areas: SK: Samtskhe-Javakheti; MM: Mtskheta-Mtianeti; SK: Shida kartli; KK: Kevmo Kartli; K: Kakheti; TB: Tbilisi District; NC: North Caucasus, GEG: Gegharot; *: means that the chronological period overlaps the transition with the following period (For highlighted individuals see SI dataset S2; SI appendix tab. S3).

During the Middle Bronze Age, we observed a wide dispersion of carbon isotope ratios from − 21.2 to − 15.2 ‰ for animals and from 20.2 to − 14.7 ‰ for humans, supporting a differential consumption of C_4_ plants according to individuals (Fig. 3, SI dataset S2, SI appendix tab. 3 to 5, fig. S3). Fourteen domestic animals (bovine, caprine and a dog) from Central Georgia and two humans, also radiocarbon dated to the MBA, from Tsaghvli and Natakhtari (north-west of Tbilisi) show isotope ratios above -17.0 ‰ that indicate a sufficient quantitative consumption of C_4_ plants or meat of animals having consumed C_4_ plants to be recorded visibly in tissue consumers (Fig. 3). Considering the area and the chronological period, this result may indicate millet consumption. In comparison to the MBA, carbon isotope ratios from the most recent periods (LBA to Early Iron Age) highlight a significant increasing consumption of millets which is more visible in humans (36 samples out of 46, with δ^13^C values from -18.8 to − 10.0 ‰, Mann–Whitney, p-value(δ^13^C) < 0,000) than in animals (13 out of 31, δ^13^C values from − 20.9 to − 13.7 ‰) (Fig. 3, SI dataset S2, SI appendix tab. 3 to 5, fig. S3). Interestingly, a variability of this consumption is observed at the human intra-populational level. In Treligorebi (Tbilisi) and Abanoskhevi (north of Tbilisi), a differential quantitative consumption of C_4_ cereal is observed between individuals (either directly consumed or from animals having eaten millet) questioning the status of this crop in the human diet. One of the main results of this study is that stable isotopic analysis is in accordance with the oldest radiocarbon dates obtained on millet grains, and that before the MBA and especially during the Kura-Araxes period (ca. 3300–3000 cal BC), no C_4_ plant consumption signal is recorded, at least on the recently analyzed sites in the northern Caucasus^39,49^ as in the South^37,50,51^.

### The place of the Caucasus in the diffusion of millets across Eurasia

Our investigation into the antiquity of millets in Caucasus has clarified their chronology in the region through both radiocarbon dating and stable isotopic analyses. This new data needs to be integrated in a wider context on the place of millet in Eurasian prehistory. Recently, several published studies have considered crop dispersals from Europe and Asia and concluded that common millet did not reach the Caucasus before 1500 BC, nor was foxtail millet present in Western Asia before 500 BC^52,53^. Several lines of evidence from our research introduce new chronological milestones and poses several questions: (1) What is the state of knowledge on the chronology of the spread of millet across Eurasia? (2) From a Caucasian perspective, from where did millet(s) arrive and when? (3) What are the implications of our research for the possible domestication of common or foxtail millet in the Caucasus?

#### From the North or the South? Spread of millet(s) across Eurasia to the Caucasus

**Figure 4:**
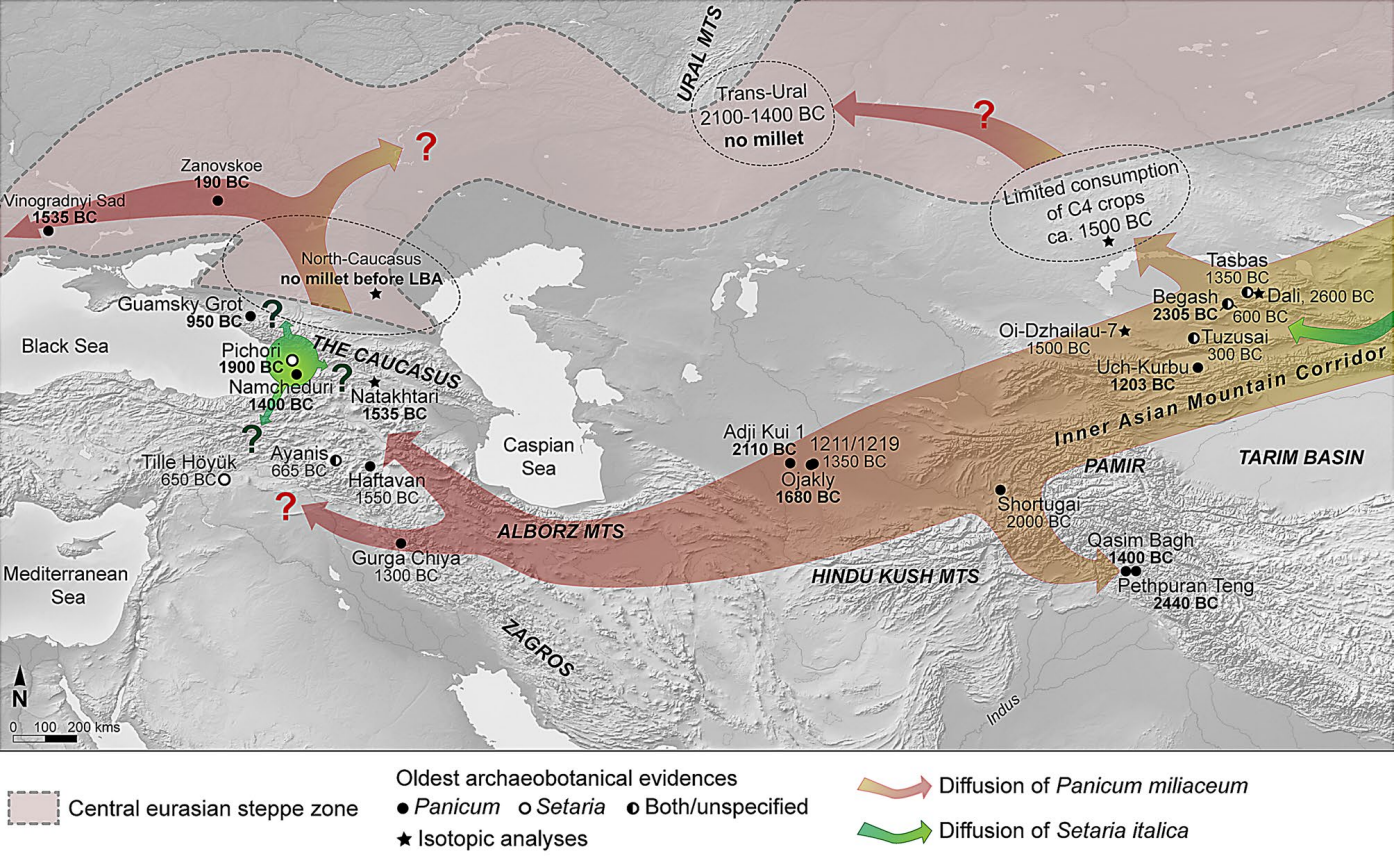
A schematic diffusion of millets (*Panicum miliaceum* and *Setaria italica*) across Eurasia and the Caucasus. **Bold dates are millet grains directly dated**. The dates are averages. E.g. “mid-2nd millennium” = 1500 BC. When 14C available: 2 sigma is preferred and rounded (e.g. for Tasbas, 1416–1287 cal BC = 1350 BC). BC implies cal BC. (Background map made with Natural Earth @ naturalearthdata.com).

The concept of “food globalization” is based on the fact that numerous economic plants have moved across Eurasia at various stages in Prehistory, such as wheat, barley, rice and millet. During the last decades, studies conducted on crop movement across Prehistoric Eurasia show that “food globalization” began in the third millennium BC and intensified during the second millennium BC^2,53^. The diffusion of millet compared to other starchy plants seems to respond more to ecological than economic and cultural factors, due to its short growth cycle and ripening period. Indeed, the expansion of millet can be characterized as driven by “risk-minimizing subsistence strategies within Asian Landscapes variously constrained by long winters and scarcity of water”^2^. Nevertheless, archaeobotanical analysis recently carried out in northern Central Asia indicate a mixed cropping system relying on cultivated plants such as free-threshing wheat, barley and pulses throughout the Bronze Age^54^. Numerous analyses—mainly in Kazakhstan and Uzbekistan—have been carried out recently to track the spread and progression of millet westwards (Fig. 4). These synthetic studies are based on both archaeobotanical and isotopic studies and indicate that the chronology of the first millet record varies according to the sites and their altitude, from the end of the third millennium to the mid-second millennium^3,5,11,12,52,55,56^. In Central Asia, several Bronze age sites have yielded millet grains situated within a region known as the *Inner Asian Mountain Corridor* (Fig. 4, ^58^) that has served as a passageway. In Southeast Kazakhstan, the site of Begash gives the earliest occurrence of broomcorn millet in Central Asia, attributed to 2280–2060 cal BC^10^. Not far from Begash, at the site of Tasbas, millet remains (grains and phytoliths of *Panicum* and *Setaria*) were dated to around 1350 BC^58^ and millet from Uch-Kurbu was directly dated to 1279–1127 cal BC^59^. In Dali, combined genetic and stable isotopic analyses of faunal remains demonstrate dietary intake with a substantial C_4_ component by 2700 BC^11^. Consumption of millet from the Middle-Late Bronze Age onwards (ca. 1800 cal BC), and likely eaten directly by humans is also shown by stable isotope studies carried out in Southern Kazakhstan with a C_4_ signal recorded in Oi-Dzhailau-7 (− 16.9 to − 13.7 ‰) around 1500 BC^55^. To the southwest, common millet (undated) at Shortugai was identified in contexts from around 2000 BC^60^, and at Qasim Bagh in Kashmir common millet was dated directly to the middle of the 2nd millennium^61^. In this same region of Indian Kashmir, it can be noted that the millets of Pethpuran Teng have recently been dated to around 2500 cal BC and are therefore older than in Begash^62^. In the Murghab region (Eastern Turkmenistan), three sites—Adji Kui1, Ojakly and 1211/1219—provided grains of *Panicum*, of which two offered direct radiocarbon dates: 1740–1614 cal BC for Ojakly^63^ and an older date in Adji Kui 1 at 2272–1961 cal BC^64^. This data reveals a chronological framework for the westward expansion of millet, mostly *Panicum*, between 2300 and 1300 BC. To the west, the data is very incomplete: there is a geographical gap between the Murghab region and the northwest of Iran (Fig. 4). In Iraq and Iran, the data is similarly quite sparse. Millets are present on some sites, but are not directly dated: at Gurga Chiya, they are in contexts dated to around 1300 BC^65^ and Haftavan Tepe to around 1550 BC^66^. The dated *Panicum* and *Setaria* grains from Namcheduri and Choloki in this study provide the missing radiocarbon evidence to chronologically connect the westward expansion of millets from China via Central Asia to the Near East and Europe.

Millet, at least common millet, probably did not arrived in the Caucasus via the Eurasian steppes. Across the Eurasian steppe zone, north of the *Inner Asian Mountain Corridor*, the evidence for millet consumption is mainly based on stable isotope studies (Fig. 4). In Central Kazakhstan, the isotopic signal that would indicate the intake of C_4_ plants is discrete and only appears at the end of the Bronze Age^55,67,68^. On sites occupied during the Bronze Age (2100–1400 BC), millet is absent, both as macro botanical remains (e.g. in Trans-Urals, ^70^] and from the stable isotope data^70^. Furthermore, a recent synthesis on the subject using stable isotope meta-analysis from Mongolia to the northern Caucasus indicates that millet was not consumed by humans or livestock in Southwestern Siberia and Trans-Urals before the Iron Age^56^. In summary, the data agrees with Jones et al.’s (2016) conclusion that the northern steppe route appears to have only became agricultural in the first millennium BC. Therefore, the distribution of millet from China to the Caucasus has tended to be from the south, following Pamir, Hindu Kush and Zagros mountains, rather than from the Eurasian steppe zone (Fig. 4).

#### On the road to Europe

In the North Caucasus, millet grains have not been identified at sites before the Iron Age, and there is no isotope signal oriented towards a consumption of millet before the Late Bronze Age (Koban culture) and the Iron Age^39,71^. In the Bronze Age Caspian and Low Don Steppe (2500–2350 cal BC), similarly no consumption of millet is registered through archaeobotanical or isotopic analyses^72^. Interestingly, at the site of Vinogradnyi Sad in western Ukraine *Panicum* was directly dated to 1622–1452 cal BC^8^, which is a little bit older than *Panicum* grains from Namcheduri. The Mid Second millennium (1600–1400 BC) is a period of explosive expansion of *Panicum* cultivation in western Eurasia, millet appears in the Caucasus, as well as in Ukraine and South-East Europe^8^. It is difficult with so few dated grains to draw a pathway of millet expansion between the Caucasus and Europe, via either the north Pontic region or Anatolia. The grains from Sos Höyük however provide the earliest directly dated evidence for millet in Anatolia (1271–1039 cal BC) which together with other undated finds of broomcorn millet in mid-late second millennium contexts at Troy^73^ and Kusakli^74^ suggest a delayed dispersal of millets over Anatolia. It is more likely therefore that *Panicum* spread westwards from the Caucasus along the northern Black Sea Coast into Ukraine (Fig. 4).

#### A possible domestication of foxtail millet in the Caucasus?

In the end, were millets domesticated in the Caucasus? Concerning *Panicum*, genetic evidence, coupled with archaeobotany and paleodietary analysis “are now consistent with a single origin of cultivated *P. miliaceum* somewhere in northern China, at least by the 6th millennium BC.”^75^. Our results converge to support the hypothesis of a single primary domestication center in East Asia and not scattered in different points of the globe, since we have no evidence of broomcorn millet dated before the 3^rd^ millennium in the Caucasus. For *Setaria*, we have a different pattern: the wild progenitor of foxtail millet—*Setaria viridis—*is widespread across Eurasia. If genetic analyses indicate a single domestication center for foxtail millet in China^76,77^ then more research is needed to understand its early presence in the Caucasus. Currently, foxtail millet does not appear in Central Asia before 1500 BC. Moreover, it only appears cultivated in quantity in Anatolia at Ayanis and Tille Höyük in the Iron Age^66,78^. *Setaria* is the only millet and the oldest dated in the Western Caucasus (Pichori, Abkazia), and it is well represented alongside *Panicum*, at slightly later sites (Namcheduri and Choloki) near Pichori, on the Black Sea coast. The ancient and very localized appearance of foxtail millet raises the question of a possible domestication of this species in the Western Caucasus. Many wild forms of *Setaria* exist in this region, especially the wild progenitor of *Setaria italica*, *Setaria viridis*^29,79^. Although it is not possible to distinguish between the C_4_ signal of *Panicum* and *Setaria,* the earliest isotopic evidence for C_4_ plant consumption are found in the southern part of the Caucasus, on two sites that are contemporary with Pichori (Tsaghvli and Natakhtari, ca. 1850–1400 cal BC). The date and spread of *Setaria* across Eurasia are still uncertain (Fig. 4). Of course, we must be cautious because the process of *Setaria* domestication, involving morphological, genetic and practical changes is incompletely understood. In the light of the ancient dates we have, *Setaria* may have been independently domesticated in the Caucasus. Whether this was a localized and shortlived cultivation of *Setaria* in the Western Caucasus or if foxtail millet spread from here to nearby regions is still hypothetical. The early presence of *Setaria* in the Caucasus opens a new avenue which needs to be investigated with further ethnobotanical^79^, archaeobotanical and genetic studies^76,77^.

## Conclusion

By combining archaeobotanical and stable isotope analyses and with the support of a large set of radiocarbon dates, we can reconsider the chronological framework and bring new elements to the model of crop dispersal in Eurasia. Our results shed a new light on the question of millet in the Caucasus: *Setaria italica* and *Panicum miliaceum* are not present in the Neolithic; their cultivation appears slightly in the Middle Bronze Age as proven by both direct millet dating and isotopic data. Isotopic analyses reveal that millets became more important in the human and animal diet during the Late Bronze Age. It is interesting that the appearance of millet in the south Caucasus coincides with important socio-economic changes. The Late Bronze Age is the period when agricultural intensification begins in this region, and is also a time of important cultural interactions between the Colchian and Koban cultures. Further research may reveal the relationship between millet cultivation and societal developments in the Late Bronze Age Caucasus. Considering the timing of millet’s spread across Eurasia, these results demonstrate that the Caucasus was not an independent domestication center for *Panicum miliaceum*. The question remains open concerning *Setaria italica*: it appears as early as 2000 cal. BC in the Western Caucasus, but much later (ca. 1500–1000 cal. BC) in other parts of the region. Since wild ancestor exist in the Caucasus, it is possible that foxtail millet was locally domesticated in the late Middle Bronze Age. Domestication and consumption of millet constitute a fundamental element in the history of the development of agriculture across Eurasia, as it is shown by recent research of food globalization.

## Materials and methods

Considering archaeobotanical (charred millet grains) and isotopic investigations, 61 sites located in Russian Federation, Georgia, Armenia, Azerbaijan and closed part from the Caucasus area in Turkey and Iran provided some material.

### Archaeobotanical material and radiocarbon dates

Considering both old and recent excavations, priority was given to macroremains that is grains, which are direct witnesses of its cultivation and collected remains of millet in Georgia, Armenia, Russian federation and North-East Turkey. No remains of millet were available in Azerbaijan. For each sample, we checked the provenance and the context, and we verified the identification. Despite carbonization and sometime bad preservation, identification of the grains as broomcorn millet and foxtail millet were clear, with a characteristic morphology: we checked their overall form, size and the position of the embryo and when available, ornamentations on their lemmas and paleas (*Setaria* sp.). Several publications have been used to verify our identifications^80,81^, in particular to differentiate between cultivated species (*Panicum miliaceum/Setaria italica*), immature grains of these species, often small in size, and grains of closely related wild species, e.g. *Setaria viridis*, *S. verticillata*, *S. glauca*, *Echinochloa crus-galli*. Grains which were sent to radiocarbon dating were pictured (SI appendix fig. S2). After completing the inventory of millet remains found in the Caucasus, we were able to recover 17 samples from archaeological contexts dated between Early Bronze Age to Antiquity, among which we choose grains for direct AMS radiocarbon dates.

### Bone and grain material, pretreatment protocol and isotopic measurements

The corpus belongs to 83 human individuals, 110 animals and two millet grains from 23 archaeological sites (SI dataset S2), in order to accurately map the ecosystems specific to each region of Georgia. Samples were chosen because of their well-controlled archaeological context. If there was any doubt about the chronology of the samples, human and animal bones were subsampled and individually radiocarbon dated to ensure the reliability of each set of data (SI appendix fig. S1; SI dataset S1). The protocol proposed by Longin^82^ and modified by Bocherens et al.^83^ was used to extract human and animal bone collagens. After grinding bone chunk into a powder (> 700 µm), samples (~ 250 mg) were soaked in HCl (1 M, 20’) for demineralization, followed by a soak in NaOH (0.125 M, < 18 h) to remove contaminants and dissolved in HCl (pH = 2, 17 h, 100 °C). Samples were then freeze-dried for 48 h. An acid–base-acid protocol as described by Bogaard et al.^84^ was used for charred grains. Samples were soaked in HCl (0.5 M, 70 °C, for 30–60 min), then rinsed to reach a neutral pH and soaked in NaOH (0.1 M, 70 °C, 60 min), rinsed again and soaked in HCl (0.5 M, 70 °C, for 30–60 min). Dried samples were ground to a fine powder. The isotopic analysis of human and animal bone collagens and charred grains was performed at the Iso-Analytical Limited laboratory (Cheshire, UK). Pre-weighed human bone collagens and plants were analyzed with a Europa Scientific elemental analyzer interfacing with a Europa Scientific 20–20 (IRMS) to yield data on elemental composition (%C, %N) and measurements of δ^13^C and δ^15^N. Isotopic measurements were run against benchmark standards (PDB for carbon, AIR for nitrogen) and expressed in parts per mil (‰). For both collagen and bulk plants, IAEA standards are bovine liver (IA-R042), a mixture of ammonium sulphate (IA-R045) and beet sugar (IA-R005) and a mixture of sugar cane (IA-R006) and ammonium sulphate (IA-R046). Measurement reproducibility of a repeat sample is of ± 0.1‰ for δ^13^C and δ^15^N values. Bone collagen preservation was checked according to several criteria, such as the yield of extraction (≥ 10 mg.g-1), the percentages of C and N (%C ≥ 30% and %N ≥ 10%) and the atomic C:N ratio (2.9 < C:N < 3.6)^85–87^.

## Supplementary Information

Supplementary Information 1.

Supplementary Information 2.

Supplementary Information 3.
