# Supplementary material for: The place of millet in food globalization during Late Prehistory as evidenced by new bioarchaeological data from the Caucasus
Source: Sci Rep. 2021 Jun 23;11:13124. doi: 10.1038/s41598-021-92392-9 (PMC8222238; doi:10.1038/s41598-021-92392-9)
Supplement: Supplementary file 1 — Supplementary Information 1. [file 41598_2021_92392_MOESM1_ESM.pdf]

Supplementary Information for

The place of millet in food globalization during Late Prehistory as evidenced by new bioarchaeological data from the Caucasus

Lucie MARTIN, Estelle HERRSCHER, Erwan MESSAGER, Giorgi BEDIANASHVILI, Nana RUSISHVILI, Elena LEBEDEVA, Catherine LONGFORD, Roman HOVSEPYAN, Liana BITAZDE, Marina CHKADUA, Nikoloz VANISHVILI, Françoise LE MORT, Kakha KAKHIANI, Mikael ABRAMISHVILI, Giorgi GOGOCHURI, Bitzina MURVANIDZE, Gela GIUNASHVILI, Vakhtang LICHELI, Aurélie SALAVERT, Guy ANDRE.

Lucie MARTIN, Estelle HERRSCHER
Email: lucie.martin@unige.ch, estelle.herrscher@univ-amu.fr

This PDF file includes:

- **Table S1:** List of sites in the Caucasus containing (i) millet grains recovered in the literature and from ancient/recent excavations;(ii) animal and human bones used for isotopic analyses.
- **Figure S1**: Calibrated radiocarbon dates made on millet grains (*Panicum miliaceum* or *Setaria italica*) in green, human (H) and animal bones (F).
- **Table S2**: Table listing the millet grain samples directly dated with detailed dates, provenance of the samples and their presumed chronology before the dates (in order of their numbering in Fig. 1).
- **Figure S2**: Photography of millets (*Panicum miliaceum* and *Setaria italica*) identified on several recent excavation and directly dated; scale: 1 mm.
- **Table S3**: Breakdown table for human and animal individuals by geographical area and chronological period.
- **Table S4**: Descriptive statistics of carbon and nitrogen isotopic ratios.
    - Table S4.1. Descriptive statistics of human carbon and nitrogen isotopic ratios according to geographical areas and chronological periods.
    - Table S4.2. Descriptive statistics of animal carbon and nitrogen isotopic ratios according to geographical areas and chronological periods.
    - Table S4.3. Descriptive statistics of animal carbon and nitrogen isotopic ratios according to animal groups (bovid, caprid), chronological periods and geographical areas.
- **Table S5**: Results of statistical tests performed on human and animal carbon and nitrogen isotope ratios using R©3.6.1.
- **Figure S3**: Box-plots of human carbon isotope ratios.
    - Figure S3.1. Box-plots of human carbon isotope ratios according to geographical areas (A), to chronological groups and geographical areas (B1: Middle Bronze Age, B2: Late Bronze Age to Late Iron Age), and human pooled together according to chronological groups (C).
    - Figure S3.2: Box-plots of animal carbon isotope ratios according to geographical areas (A), to chronological groups and geographical areas (B1: Middle Bronze Age, B2: Late Bronze Age to Late Iron Age), and human pooled together according to chronological groups (C)
    - Figure S3.3. Box-plots of animal carbon isotope ratios according to chronological groups for bovid (A) and caprid (B).
- **SI references**

SI appendix Table S1

Fig. 1, site numbers	Name of the site	Country, Region/Province, District/Municipality	Period of occupation (according to archaeological data)	Analyses: Archaeo-botany/ Isotopes/ Both	Human/ Fauna/ Plant	Material directly 14C dated	Isotopes: number of **human** bone sample analyzed	Isotopes: number of **animal** bone sample analyzed	Reference number
**RUSSIAN FEDERATION**									
1	Chishkho	Adygea,Teuchezhsky	MBA	Archbot	P	yes	-	-	1
2	Lesnoe	Adygea, Maykop	LBA	Archbot	P	yes	-	-	1
3	Guamsky Grot	Krasnodar Krai, Apsheronsky	LBA	Archbot	P	yes	-	-	2
4	Kislodovsk	Stavropol Krai	IA	Isotope	H	no	1	-	3
5	Kabardinka-2	Stavropol Krai, Predgorny	LBA	Archbot	P	yes	-	-	4
6	Koban	North Ossetia	(E)IA	Isotope	H/F	yes	7	3	3, 5, 6
7	Chidgom	North Ossetia, Alagir	EBA/MBA	Archbot	P	yes	-	-	7, 8
8	Velikent I	Dagestan, Derbent district	EBA (2500-2000 BC)	Archbot	P	yes	-	-	9
**GEORGIA**									
9	Etseri village	Samegrelo-Zemo Svaneti	EIA (1st mill BC)	Archbot	P	no	-	-	10
10	Pichori	Abkhazia	MBA/LBA	Archbot	P	yes	-	-	11, 12 (p. 454-456)
11	Dikha-Gudzuba	Samegrelo-Zemo Svaneti, Zugdidi	VII-VIth cent. BC	Archbot	P	no	-	-	11
12	Nosiri Megrelia	Samegrelo-Zemo Svaneti, Senaki	MBA/LBA (1900-900 BC)	Archbot	P	no	-	-	11
13	Gabashvilis Gora	Imereti, Kutaisi	EIA (800-600 BC)	Archbot	P	no	-	-	11
14	Vani	Imereti	3rd – 1st centuries BC	Archbot	P	yes		-	11, 13
15	Choloki	Adjara, Kobuleti	14-12th centuries BC	Archbot	P	yes	-	-	14
16	Namcheduri	Adjara, Kobuleti	MBA (14-13th cent. BC)	Archbot	P	yes	-	-	11, 14
17	Tsagvli	Shida Kartli	MBA	Isotope	H/F	yes	4	2	15
18	Natsargora	Shida Kartli, Khashuri	LBA	Archbot	P	no	-	-	11, 16, 17
19	Gudabertka	Shida Kartli, Gori	EBA (Kura-Araxes)	Archbot	P	yes	-	-	11, 18
20	Grakliani Gora	Shida Kartli, Kaspi	LBA	Both	H/P	yes	3	-	19
21	Abanoskhevi	Mtskheta-Mtianeti	MBA/LBA	Isotope	H/F	yes	6	17	20, 12 (p. 203, 207)
22	Tsikhia Gora	Shida Kartli, Kaspi	MBA/LBA	Archbot	P	no	-	-	11
23	Tsitsamuri	Mtskheta-Mtianeti, Mtskheta	MBA	Isotope	F	yes	-	10	21
24	Natakhtari	Mtskheta-Mtianeti, Mtskheta	MBA	Isotope	H/F	yes	6	7	22, 23, 24
25	Samtavro (settlement)	Mtskheta-Mtianeti, Mtskheta	MBA	Isotope	H/F	yes	5	14	2, 25, 12 (p. 417-418)
26	Narekvavi	Mtskheta-Mtianeti, Mtskheta	IA (7th-4th cent. BC)	Archbot	P	yes	-	-	11, 26
27	Treli/Treligorebi	Tbilisi	LBA/IA	Both	H/F/P	yes	13	13	27, 12 (p. 417-418)
28	Digomi	Tbilisi	LBA	Archbot	P	no	-	-	11
29	Atskuri	Samtskhe-Javakheti	MBA	Isotope	H/F	yes	11	31	28, 29
30	Tiselis Seri	Samtskhe-Javakheti	EBA (Kura-Araxes)	Archbot	P	no	-	-	30
31	Akhchia	Samtskhe-Javakheti	MBA	Isotope	H	no	1	-	31
32	Bertkama- Chalis kurgan	Samtskhe-Javakheti	MBA	Isotope	H	yes	19	-	31
33	Chala	Kvemo Kartli, Algeti National Park	LBA II	Isotope	H	no	1	-	32, 33
34	Kobala	Kvemo Kartli, Algeti National Park	LBA I	Isotope	H	no	2	-	32
35	Irganchai, Tashiri	Kvemo Kartli, Dmanisi	LBA I	Isotope	H	no	1	-	34, 35
36	Karataki	Kvemo Kartli, Dmanisi	MBA	Isotope	F	yes	-	9	Unpublished
37	Gantiadi	Kvemo Kartli, Dmanisi	MBA	Isotope	H/F	yes	1	4	34
38	Dalari	Kvemo Kartli, Dmanisi	LBA II	Isotope	H	no	1	-	36
39	Aruchlo 1	Kvemo Kartli, Bolnisi	5th–4th mill. BC, Late Neolithic	Archbot	P	no	-	-	37
40	Imiris-Gora	Kvemo Kartli, Marneuli	5th–4th mill. BC, Chalcolithic	Archbot	P	no	-	-	10, 38
41	Khramebi	Kakheti, Nukriani	MBA	Isotope	H	no	1	-	39
42	Noname-Gora	Kakheti	IA (8th-7th cent. BC)	Archbot	P	yes	-	-	40, 41
43	Ciskaraant Gora	Kakheti, Dedoplistskaro	EBA/MBA (3rd mill. BC)	Archbot	P	no	-	-	40, 42
**ARMENIA**									
44	Sev-Sev Kareri Blur	Tavush	EIA (1st mill. BC)	Archbot	P	no	-	-	10
45	Yenokavan-2	Tavush, Yenokavan	EIA (1st mill. BC)	Archbot	P	no	-	-	43
46	Gegharot	Aragatsotn, Gegharot	LBA	Archbot	P	yes	-	-	44, 45
47	Tsaghkahovit	Aragatsotn, Tsaghkahovit	LBA	Archbot	P	yes	-	-	45
48	Nerkin Naver	Aragatsotn, Ashtarak	MBA (XXI-XIX Cent BC)	Archbot	P	no	-	-	10, 46
49	Argishtikhinili	Armavir, Nor Armavir	LBA	Archbot	P	no	-	-	10, 47
50	Karmir Blur	Yerevan	Urartian (VII-VI centuries BC)	Archbot	P	no	-	-	10, 48
51	Aramus	Kotayk	Urartian (1st mill BC)	Archbot	P	no	-	-	10, 49, 50
**AZERBAJIAN**									
52	Mingechevir	Mingechevir	MBA, LBA, EIA	Archbot	P	no	-	-	10
53	Uzerlik-Tepe	Aghdam	MBA (2000-1500 BC)	Archbot	P	no	-	-	10, 51, 52
54	Khojaly	Askeran	EIA (end 2nd mill. BC)	Archbot	P	no	-	-	10
55	Kültepe 1	Nakhchivan, Babek Rayon	5th–4th mill. BC, Late Neolithic	Archbot	P	no	-	-	10, 12 (p. 104)
**TURKEY**									
56	Sos Höyük	Erzurum, Yiğittaşı	EBA (Kura Araxes), MBA, EIA	Archbot	P	yes	-	-	53
57	Ayanis	Van, Ağarti	IA, Urartian (685–645 BC)	Archbot	P	no	-	-	54
**IRAN**									
58	Haftavan Tepe	West Azerbaijan, Urmian basin	1550 BC	Archbot	P	no	-	-	55
**Total of human/animal bone samples for isotopic analyses**							**83**	**110**	

EBA: Early Bronze Age; MBA: Middle Bronze Age; LBA: Late Bronze Age; IA: Iron Age; EIA: Early Iron Age

Table S1: List of sites in the Caucasus containing (i) millet grains recovered in the literature and from ancient/recent excavations; (ii) animal and human bones used for isotopic analyses.
For each site, we indicate:
- its localization;
- the "presumed" period of occupation, according to archaeological data;
- the type of analyses used (archaeobotany and/or isotopes);
- the types of remains present and/or analyzed (human bone, animal bone or plant remains);
- if radiocarbon dates were directly made on material (see figure and dataset S1 for details);
- the number of human and animal bones sampled for isotopic analyses and references.
- the reference number (bibliography at the end of SI)

SI appendix Figure S1

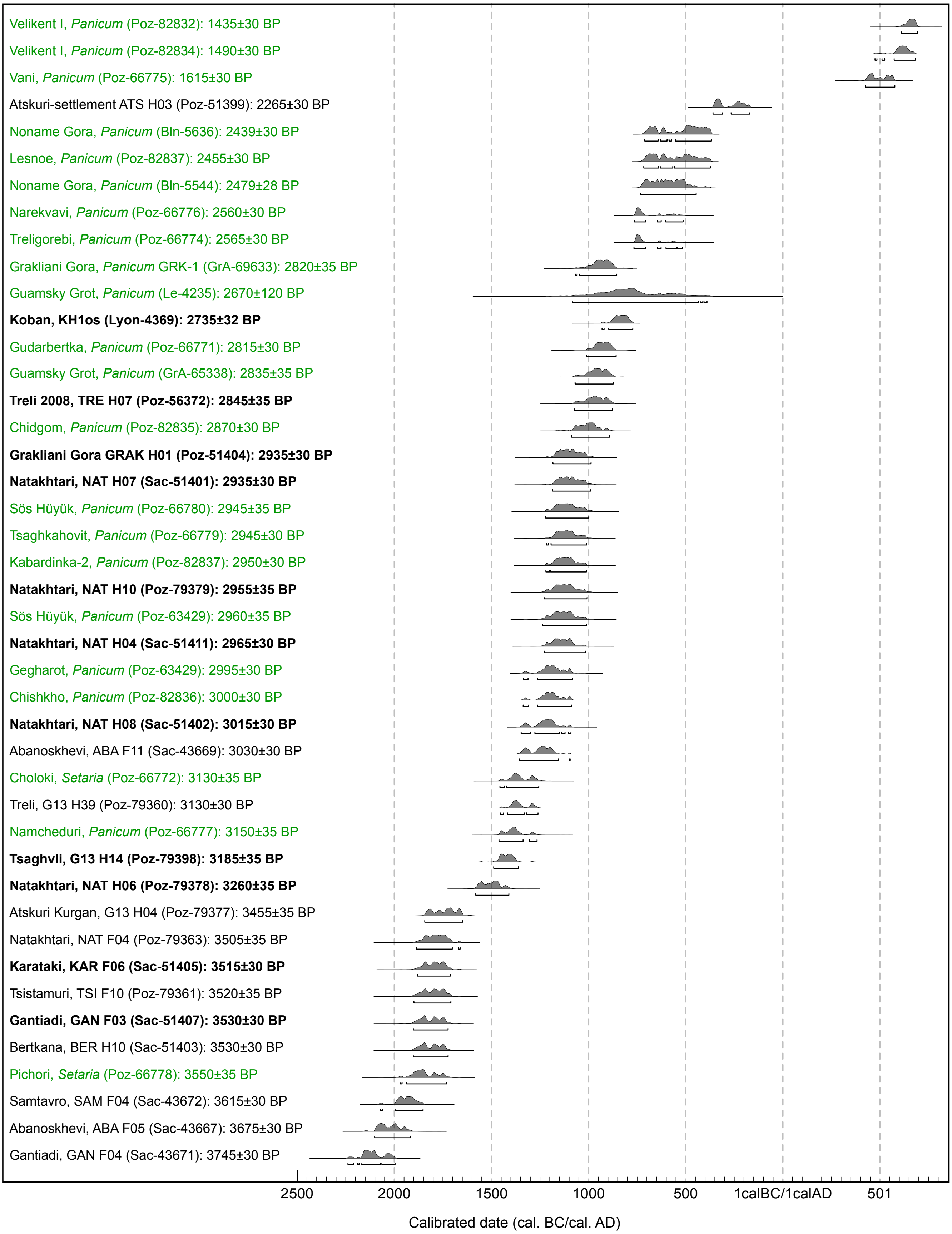

Figure S1: Calibrated radiocarbon dates made on millet grains (*Panicum miliaceum* or *Setaria italica*) in green, human (H) and animal bones (F).
In bold = human or animal subject with isotopic contents characteristic of C4 plant consumption (d13C≤17‰).
Realised with OxCal v4.3.2 ; r:5
IntCal13 atmospheric curve

References 56 and 57

SI Appendix Table S2

Fig.1	Site	Species dated	N° sample, provenance	Datation and/or culture (presumed)	Code lab	Date BP	cal BC 95.4% (2 sigma)	Period according to 14C dates
1	Chishkho	*Panicum miliaceum*	1801	MBA	Poz-82836	3000 ± 30 BP	1377-1126 cal BC	LBA
2	Lesnoe	*Panicum miliaceum*	1809	LBA	Poz-82837	2455 ± 30 BP	756-413 cal BC	IA
3	Guamsky Grot	*Panicum miliaceum*	Layer 4/5, square 69/70–86/87, Fireplace 2	LBA	GrA-65338	2835 ± 35 BP	1110-908 cal BC	LBA
3	Guamsky Grot	*Panicum miliaceum*	Layer 4/5, square 69/70–86/87, Fireplace 2	LBA	Le-4235	2670 ± 120 BP	1124-430 cal BC	LBA/IA
5	Kabardinka-2	*Panicum miliaceum*	2332 (square 2, crack), accumulation of approx. 100 seeds	LBA	Poz-82838	2950 ± 30 BP	1260-1051 cal BC	LBA
7	Chidgom	*Panicum miliaceum*	2317, 17 samples of 10 litres each, flottation, only 2 grains of millet	EBA/MBA	Poz-82835	2870 ± 30 BP	1127-931 cal BC	IA
8	Velikent I	*Panicum miliaceum*	2173, unit 0-1, locus 7	EBA (2500-2000 BC)	Poz-82832	1435 ± 30 BP	571-655 AD	Late Antique
8	Velikent I	*Panicum miliaceum*	2176, unit 0, locus 4	EBA (2500-2000 BC)	Poz-82834	1490 ± 30 BP	436-644 AD	Late Antique
10	Pichori	*Setaria italica*	2nd t / ash layer	MBA/LBA	Poz-66778	3550 ± 35 BP	2011-1771 cal BC	MBA
14	Vani	*Panicum miliaceum*	Area 8th, In a pot, "millet cake"	IA (3rd–1st cent. BC)	Poz-66775	1615 ± 30 BP	387-432 AD	Antique
15	Choloki	*Setaria italica*	Vth layer, millet clustered	LBA (14th-12th cent. BC)	Poz-66772	3130 ± 35 BP	1496-1296 cal BC	LBA
16	Namcheduri	*Panicum miliaceum*	// layer 5, square 9, section SW, "20 cm of charred seeds"	MBA	Poz-66777	3150 ± 35 BP	1501-1305 cal BC	MBA
19	Gudabertka	*Panicum miliaceum*	unknown	EBA (Kura-Araxes)	Poz-66771	2815 ± 30 BP	1052-898 cal BC	LBA/IA
20	Grakliani Gora	*Panicum miliaceum*	Pot full of grains.	LBA	GrA-69633	2820 ± 35 BP	1108-896 cal BC	LBA/IA
26	Narekvavi	*Panicum miliaceum*	Kalandadris Gora W2, house 15	IA (7th-4th cent. BC)	Poz-66776	2560 ± 30 BP	805-553 cal BC	IA
27	Treligorebi	*Panicum miliaceum*	Inside the oven	LBA/IA	Poz-66774	2565 ± 30 BP	806-556 cal BC	IA
42	Noname-Gora	*Panicum miliaceum*	Burned house (H10-b9, 2G 97)	IA (8th-7th cent. BC)	Bln-5636	2439 ± 30 BP	751-408 cal BC	IA
42	Noname-Gora	*Panicum miliaceum*	Burned house 233 gr. (GI, H16-33 NG, 1999)	IA (8th-7th cent. BC)	Bln-5544	2479 ± 28 BP	772-486 cal BC	IA
46	Gegharot	*Panicum miliaceum*	Ar/Ge.T32.Δ41.2011	LBA	Poz-63429	2995 ± 30 BP	1376-1122 cal BC	LBA
47	Tsaghkahovit	*Panicum miliaceum*	Ar/Ts.SLT.11.13 (s.1)	LBA	Poz-66779	2945 ± 30 BP	1258-1049 cal BC	LBA
56	Sos Hüyük	*Panicum miliaceum*	SOS26. M16 36 86 81 S.192	EBAI/II (3000-2500 BC)	Poz-66780	2945 ± 35 BP	1261-1039 cal BC	LBA
56	Sos Hüyük	*Panicum miliaceum*	SOS64. L16c 40 35 71 S.188	MBAII (2000-1500 BC)	Poz-66781	2960 ± 35 BP	1276-1051 cal BC	LBA

EBA: Early Bronze Age; MBA: Middle Bronze Age; IA: Iron Age

Table S2: Table listing the millet grain samples directly dated with detailed dates, provenance of the samples and their presumed chronology before the dates (in order of their numbering in **Fig. 1**)

SI appendix Figure S2

Photography of millets (*Panicum miliaceum* and *Setaria italica*) identified on several recent excavation and directly dated; scale: 1 mm.

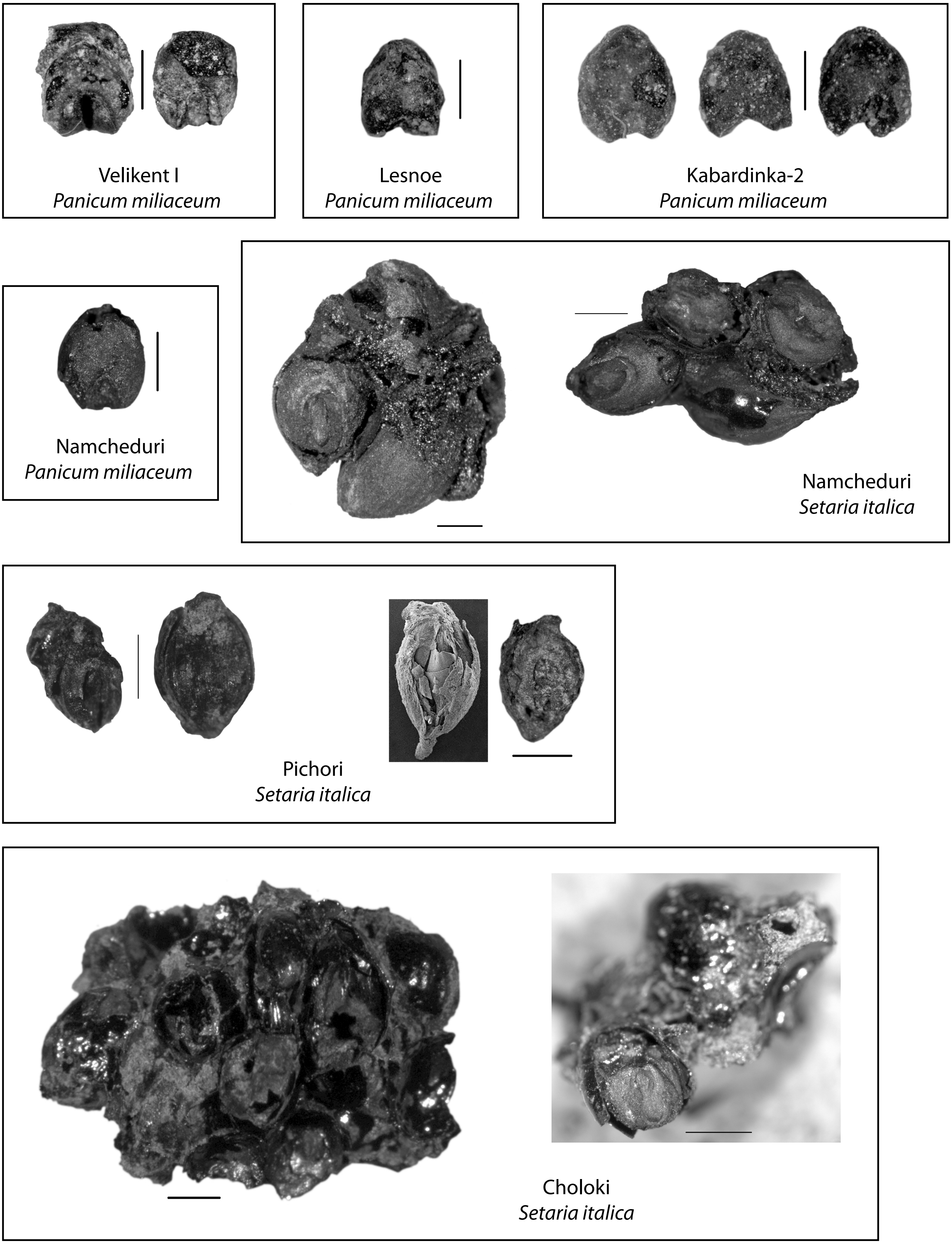

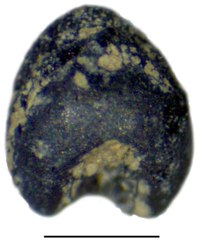

Treli
Panicum miliaceum

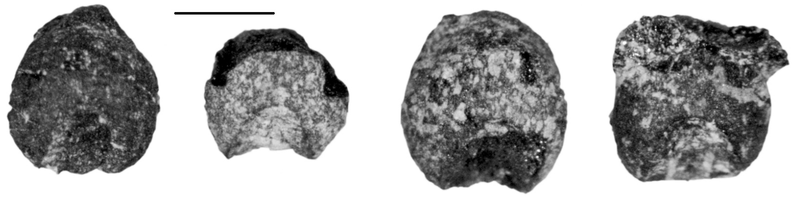

Gudabertka
Panicum miliaceum

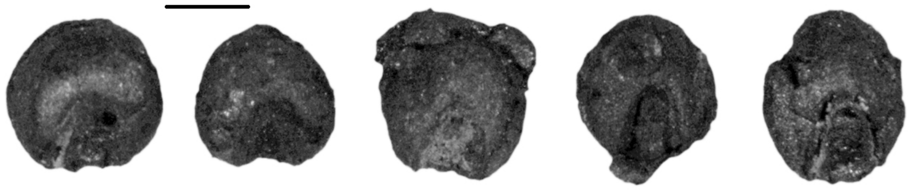

Narekvavi
Panicum miliaceum

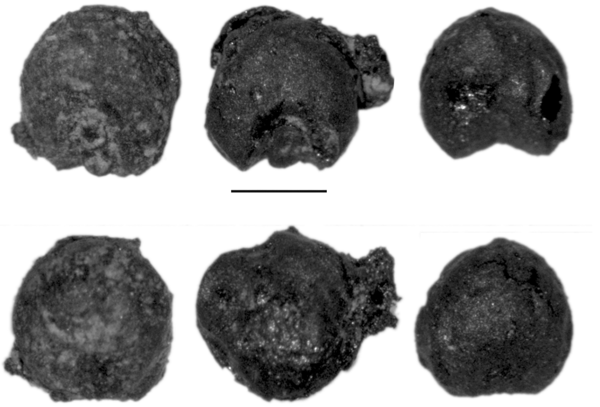

Gegharot
Panicum miliaceum

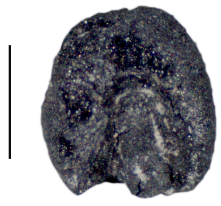

Tsaghahovit
Panicum miliaceum

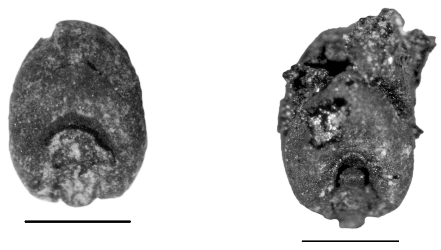

Sös Hüyük
Panicum miliaceum

SI appendix Breakdown table for human and animal individuals by geographical area and chronological period

Table S3

	Middle Bronze Age										Late Bronze Age to Iron Age							
	Bovid	Canid	Caprid	Equid	Fowl	Suid	WHerb.	Sm	Human	TOTAL	Bovid	Caprid	Equid	Suid	WHerb.	Human	TOTAL	TOTAL
**Kakheti**									**1**	**1**								**39**
Khramebi village Nukriani									1	1								**1**
**Kvemo kartli**	**4**	**1**	**7**				**1**			**13**						**6**	**6**	**19**
Chala, Algeti																1	1	**1**
Dalari																1	1	**1**
Gantiadi	1		2				1			4						1	1	**5**
Karataki	3	1	5							9								**9**
Kobala																2	2	**2**
Tashiri, Irgan-chai																1	1	**1**
**Mtskheta-Mtianeti**	**13**		**16**			**1**	**1**		**1**	**32**	**6**	**9**	**2**			**16**	**33**	**65**
Abanoskhevi	2		5			1				8	3	4	2			6	15	**23**
Natakhtari	1		6						1	8						5	5	**13**
Samtavro	3		2				1			6	3	5				5	13	**19**
Tsitsamuri	7		3							10								**10**
**North Caucasus**												**2**		**1**		**8**	**11**	**11**
Kislodovsk																1	1	**1**
Koban												2		1		7	10	**10**
**Samtskhe-Javakheti**	**10**		**9**	**1**	**1**	**9**		**1**	**30**	**61**						**1**	**1**	**62**
Akhchia									1	1								**1**
Atskuri	10		9	1	1	9		1		31								**31**
Atskuri Kurgan									10	10								**10**
Atskuri settlement																1	1	**1**
Bertkama (Chalis kurgan)									19	19								**19**
**Shida kartli**			**2**						**4**	**6**						**3**	**3**	**9**
Grakliani																3	3	**3**
Tsaghvli			2						4	6								**6**
**Tbilisi District**											**6**	**5**		**1**	**1**	**13**	**26**	**26**
Treli																13	13	**13**
Treligorebi											6	5		1	1		13	**13**
**TOTAL**	**27**	**1**	**34**	**1**	**1**	**10**	**2**	**1**	**36**	**113**	**12**	**16**	**2**	**2**	**1**	**47**	**80**	**193**

Sm: Small game

WHerb.: Wild herbivore

SI appendix

Table S4.1

Descriptive statistics of human carbon and nitrogen isotopic ratios according to geographical areas and chronological periods

	δ13C (‰)									δ15N (‰)								
	n	min	Q1	med	Q3	max	IQR	mean	sd	n	min	Q1	med	Q3	max	IQR	mean	sd
**Human**	**83**	**-20,2**	**-19,1**	**-17,4**	**-16,0**	**-10,0**	**3,1**	**-17,3**	**2,0**	**83**	**6,8**	**8,1**	**9,4**	**10,2**	**12,7**	**2,1**	**9,3**	**1,4**
Animal	110	-21,2	-19,4	-18,3	-17,3	-13,7	2,1	-18,1	1,6	110	2,1	5,0	5,9	6,7	8,5	1,8	5,8	1,3
Plant	1	-10,5	--	--	--	--	--	--	--	1	7,3	--	--	--	--	--	--	--

**TOTAL**	δ13C (‰)									δ15N (‰)								
	n	min	Q1	med	Q3	max	IQR	mean	sd	n	min	Q1	med	Q3	max	IQR	mean	sd
**Human**	**83**	**-20,2**	**-19,1**	**-17,4**	**-16,0**	**-10,0**	**3,1**	**-17,3**	**2,0**	**83**	**6,8**	**8,1**	**9,4**	**10,2**	**12,7**	**2,1**	**9,3**	**1,4**
Samtskhe-Javakheti	31	-20,2	-19,4	-19,2	-18,9	-17,3	0,4	-19,2	0,5	31	6,9	7,5	8,1	8,3	11,3	0,8	8,2	0,9
Akhchia	1	-19,1	--	--	--	--	--	--	--	1	10,2	--	--	--	--	--	--	--
Atskuri Kurgan	10	-19,9	-19,4	-19,3	-19,1	-18,8	0,3	-19,3	0,3	10	7,2	7,7	8,2	8,5	9,3	0,8	8,2	0,7
Atskuri settlement	1	-17,3	--	--	--	--	--	--	--	1	8,9	--	--	--	--	--	--	--
Bertkama (Chalis kurgan)	19	-20,2	-19,3	-19,2	-18,9	-18,6	0,5	-19,2	0,4	19	6,9	7,4	8,0	8,2	11,3	0,7	8,0	1,0
Kakheti	1	-17,6	--	--	--	--	--	--	--	1	12,7	--	--	--	--	--	--	--
Khramebi village Nukriani	1	-17,6	--	--	--	--	--	--	--	1	12,7	--	--	--	--	--	--	--
Kvemo Kartli	6	-18,8	-17,2	-16,2	-16,0	-15,8	1,1	--	--	6	6,8	8,4	9,3	9,9	12,2	1,5	--	
Chala, Algeti	1	-16,1	--	--	--	--	--	--	--	1	8,8	--	--	--	--	--	--	--
Dalari	1	-15,8	--	--	--	--	--	--	--	1	9,9	--	--	--	--	--	--	--
Gantiadi	1	-17,5	--	--	--	--	--	--	--	1	9,9	--	--	--	--	--	--	--
Kobala	2	-16,2	-16,2	-16,1	-16,1	-16,0	0,1	-16,1	0,2	2	6,8	7,2	7,6	7,9	8,3	0,8	7,6	1,1
Tashiri, Irgan-chai	1	-18,8	--	--	--	--	--	--	--	1	12,2	--	--	--	--	--	--	--
Mtskheta-Mtianeti	17	-18,7	-17,1	-16,1	-14,8	-13,4	2,4	-16,1	1,5	17	7,3	9,3	9,9	10,7	11,0	1,4	9,8	1,1
Abanoskhevi	6	-17,7	-16,9	-15,9	-15,0	-14,7	1,8	--	--	6	8,9	9,9	10,0	10,3	10,7	0,4	--	--
Natakhtari	6	-16,3	-15,3	-15,0	-14,7	-13,4	0,6	--	--	6	9,4	10,3	10,8	10,9	11,0	0,6	--	--
Samtavro	5	-18,7				-16,3				5	7,3	--	--	--	9,9	--	--	--
North Caucasus	8	-16,5	-16,2	-15,7	-14,7	-10,0	1,5	-14,8	2,3	8	7,0	9,5	9,6	9,9	10,4	0,4	--	--
Kislodovsk	1	-15,6	--	--	--	--	--	--	--	1	10,4	--	--	--	--	--	--	--
Koban	7	-16,5	-16,3	-15,9	-13,9	-10,0	2,3	-14,7	2,5	7	7,0	9,5	9,5	9,7	10,1	0,2	--	--
Shida Kartli	7	-19,5	-19,1	-16,7	-16,2	-15,4	2,9	-17,5	1,7	7	8,6	9,3	10,9	11,3	11,7	2,0	10,3	1,2
Grakliani	3	-16,7	--	--	--	-15,4	--	--	--	3	8,6	--	--	--	11,5	--	--	--
Tsaghvli	4	-19,5	--	--	--	-16,4	--	--	--	4	9,2	--	--	--	11,7	--	--	--
Tbilisi district	13	-18,7	-16,8	-16,6	-15,7	-14,8	1,1	-16,4	1,1	13	9,4	9,8	10,3	11,1	12,2	1,3	10,5	0,8
Treli	13	-18,7	-16,8	-16,6	-15,7	-14,8	1,1	-16,4	1,1	13	9,4	9,8	10,3	11,1	12,2	1,3	10,5	0,8

**Middle Bronze Age**	δ13C (‰)									δ15N (‰)								
	n	min	Q1	med	Q3	max	IQR	mean	sd	n	min	Q1	med	Q3	max	IQR	mean	sd
**Human**	**36**	**-20,2**	**-19,4**	**-19,2**	**-18,9**	**-14,7**	**0,5**	**-19,0**	**1,0**	**36**	**6,9**	**7,8**	**8,2**	**9,1**	**12,7**	**1,4**	**8,6**	**1,4**
Samtskhe-Javakheti	30	-20,2	-19,4	-19,2	-19,0	-18,6	0,4	-19,2	0,4	30	6,9	7,5	8,1	8,3	11,3	0,7	8,2	0,9
Akhchia	1	-19,1	--	--	--	--	--	--	--	1	10,2	--	--	--	--	--	--	--
Atskuri Kurgan	10	-19,9	-19,4	-19,3	-19,1	-18,8	0,3	-19,3	0,3	10	7,2	7,7	8,2	8,5	9,3	0,8	8,2	0,7
Bertkama (Chalis kurgan)	19	-20,2	-19,3	-19,2	-18,9	-18,6	0,5	-19,2	0,4	19	6,9	7,4	8,0	8,2	11,3	0,7	8,0	1,0
Kakheti	1	-17,6	--	--	--	--	--	--	--	1	12,7	--	--	--	--	--	--	--
Khramebi village Nukriani	1	-17,6	--	--	--	--	--	--	--	1	12,7	--	--	--	--	--	--	--
Mtskheta-Mtianeti	1	-14,7	--	--	--	--	--	--	--	1	10,9	--	--	--	--	--	--	--
Natakhtari	1	-14,7	--	--	--	--	--	--	--	1	10,9	--	--	--	--	--	--	--
Shida Kartli	4	-19,5	-19,2	-19,1	-18,4	-16,4	0,9	-18,5	1,4	4	9,2	--	--	--	11,7	--	--	--
Tsaghvli	4	-19,5	-19,2	-19,1	-18,4	-16,4	0,9	-18,5	1,4	4	9,2	--	--	--	11,7	--	--	--

**Late Bronze Age**	δ13C (‰)									δ15N (‰)								
	n	min	Q1	med	Q3	max	IQR	mean	sd	n	min	Q1	med	Q3	max	IQR	mean	sd
**Human**	**47**	**-18,8**	**-16,9**	**-16,2**	**-15,4**	**-10,0**	**1,5**	**-16,1**	**1,6**	**47**	**6,8**	**9,4**	**9,9**	**10,6**	**12,2**	**1,2**	**9,8**	**1,2**
Samtskhe-Javakheti	1	-17,3	--	--	--	--	--	--	--	1	8,9	--	--	--	--	--	--	--
Atskuri settlement	1	-17,3	--	--	--	--	--	--	--	1	8,9	--	--	--	--	--	--	--
Kvemo Kartli	6	-18,8	-17,2	-16,2	-16,0	-15,8	1,1	-16,7	1,2	6	6,8	8,4	9,3	9,9	12,2	1,5	--	
Chala, Algeti	1	-16,1	--	--	--	--	--	--	--	1	8,8	--	--	--	--	--	--	--
Dalari	1	-15,8	--	--	--	--	--	--	--	1	9,9	--	--	--	--	--	--	--
Gantiadi	1	-17,5	--	--	--	--	--	--	--	1	9,9	--	--	--	--	--	--	--
Kobala	2	-16,2	-16,2	-16,1	-16,1	-16,0	0,1	-16,1	0,2	2	6,8				8,3	0,8	--	
Tashiri, Irgan-chai	1	-18,8	--	--	--	--	--		--	1	12,2	--	--	--	--	--	--	--
Mtskheta-Mtianeti	16	-18,7	-17,2	-16,2	-15,1	-13,4	2,1	-16,2	1,5	16	7,3	9,2	9,9	10,4	11,0	1,2	9,7	1,1
Abanoskhevi	6	-17,7	-16,9	-15,9	-15,0	-14,7	1,8	--	--	6	8,9	9,9	10,0	10,3	10,7	0,4	--	--
Natakhtari	5	-16,3	--	--	--	-13,4	--	--	--	5	9,4	--	--	--	11,0	--	--	--
Samtavro	5	-18,7	--	--	--	-16,3	--	--	--	5	7,3	--	--	--	9,9	--	--	--
North Caucasus	8	-16,5	-16,2	-15,7	-14,7	-10,0	1,5	-14,8	2,3	8	7,0	9,5	9,6	9,9	10,4	0,4	--	--
Kislodovsk	1	-15,6	--	--	--	--	--	--	--	1	10,4	--	--	--	--	--	--	--
Koban	7	-16,5	-16,3	-15,9	-13,9	-10,0	2,3	--	--	7	7,0	9,5	9,5	9,7	10,1	0,2	--	--
Shida Kartli	3	-16,7	--	--	--	-15,4	--	--	--	3	8,6	--	--	--	11,5	--	--	--
Grakliani	3	-16,7	--	--	--	-15,4	--	--	--	3	8,6	--	--	--	11,5	--	--	--
Tbilisi district	13	-18,7	-16,8	-16,6	-15,7	-14,8	1,1	-16,4	1,1	13	9,4	9,8	10,3	11,1	12,2	1,3	10,5	0,8
Treli	13	-18,7	-16,8	-16,6	-15,7	-14,8	1,1	-16,4	1,1	13	9,4	9,8	10,3	11,1	12,2	1,3	10,5	0,8

min: minimum; med: median; max: maximum; ; sd: standard deviation

Q1: first quartile (the lowest 25% of numbers); Q3: third quartile (the 51% to 75% above the median); IQR: interquartile range

SI appendix

Table S4.2

Descriptive statistics of animal carbon and nitrogen isotopic ratios according to geographical areas and chronological periods

	δ13C (‰)									δ15N (‰)								
	n	min	Q1	med	Q3	max	IQR	mean	sd	n	min	Q1	med	Q3	max	IQR	mean	sd
**Animals**	**110**	**-21,2**	**-19,4**	**-18,3**	**-17,3**	**-13,7**	**2,1**	**-18,1**	**1,6**	**110**	**2,1**	**5,0**	**5,9**	**6,7**	**8,5**	**1,8**	**5,8**	**1,3**

**TOTAL**	δ13C (‰)									δ15N (‰)								
	n	min	Q2	med	Q4	max	IQR	mean	sd	n	min	Q2	med	Q4	max	IQR	mean	sd
Bovid	39	-20,0	-18,4	-17,8	-16,4	-13,8	2,0	-17,5	1,5	39,0	3,4	4,6	5,3	6,2	8,0	1,5	5,5	1,1
Canid	1	-15,8	--	--	--	--	--	--	--	1,0	8,1	--	--	--	--	--	--	--
Caprid	50	-20,8	-19,4	-18,8	-17,7	-13,7	1,7	-18,4	1,5	50,0	2,1	4,9	6,0	6,8	8,5	1,9	5,8	1,4
Equid	3	-20,9	--	--	--	-18,2	--	--	--	3,0	4,6	--	--	--	5,9	--	--	--
Fowl	1	-17,9	--	--	--	--	--	--	--	1,0	8,1	--	--	--	--	--	--	--
Small game	1	-20,0	--	--	--	--	--	--	--	1,0	6,0	--	--	--	--	--	--	--
Suid	12	-21,2	-19,6	-19,0	-18,0	-17,8	1,6	-19,0	1,1	12,0	4,8	6,2	6,6	7,0	8,0	0,8	6,6	0,9
Wild herbivore	3	-20,4	--	--	--	-14,7	--	--	--	3,0	3,9	--	--	--	7,7	--	--	--

**Fauna by area/district**	δ13C (‰)									δ15N (‰)								
	n	min	Q1	med	Q3	max	IQR	mean	sd	n	min	Q1	med	Q3	max	IQR	mean	sd
Samtskhe-Javakheti	31	-20,0	-19,4	-18,5	-18,0	-16,6	1,4	-18,6	0,9	31,0	2,1	4,1	4,8	6,2	8,1	2,1	5,2	1,6
Atskuri	31	-20,0	-19,4	-18,5	-18,0	-16,6	1,4	-18,6	0,9	31,0	2,1	4,1	4,8	6,2	8,1	2,1	5,2	1,6
Kvemo Kartli	13	-20,0	-18,9	-18,2	-16,6	-15,5	2,3	-17,8	1,5	13,0	5,8	6,2	6,7	7,0	8,1	0,8	6,7	0,7
Gantiadi	4	-19,4	-19,0	-18,5	-17,5	-15,5	1,5	-18,0	1,7	4,0	6,2	--	--	--	7,1	--	--	--
Karataki	9	-20,0	-18,8	-17,6	-16,6	-15,8	2,2	-17,7	1,5	9,0	5,8	6,1	6,2	7,0	8,1	0,9	--	--
Mtskheta-Mtianeti	48	-21,2	-19,3	-18,3	-17,1	-13,9	2,2	-18,1	1,7	48,0	3,9	5,2	5,7	6,2	8,5	1,0	5,8	1,0
Abanoskhevi	17	-21,2	-19,4	-18,5	-18,1	-16,8	1,3	-18,8	1,2	17,0	4,2	5,2	5,5	6,0	7,7	0,8	5,7	0,8
Natakhtari	7	-18,4	-18,1	-17,9	-17,6	-16,4	0,6	-17,7	0,7	7,0	5,7	6,6	6,8	7,3	8,5	0,7	--	--
Samtavro	14	-20,4	-19,7	-18,9	-17,4	-13,9	2,3	-18,2	2,1	14,0	3,9	4,5	5,6	6,2	7,6	1,7	5,5	1,2
Tsitsamuri	10	-19,2	-17,7	-16,4	-15,6	-15,2	2,1	-16,8	1,5	10,0	4,4	5,3	5,8	6,1	6,2	0,8	5,6	0,6
North Caucasus	3	-20,8	-20,6	-20,4	-20,4	-20,3	0,2	-20,5	0,3	3,0	4,1	--	--	--	6,8	--	--	--
Koban	3	-20,8	-20,6	-20,4	-20,4	-20,3	0,2	-20,5	0,3	3,0	4,1	--	--	--	6,8	--	--	--
Shida Kartli	2	-19,3	-19,2	-19,1	-19,0	-18,8	0,2	-19,1	0,3	2,0	5,8	--	--	--	7,9	--	--	--
Tsaghvli	2	-19,3	-19,2	-19,1	-19,0	-18,8	0,2	-19,1	0,3	2,0	5,8	--	--	--	7,9	--	--	--
Tbilisi district	13	-20,4	-18,3	-16,8	-15,4	-13,7	2,9	-16,8	2,1	13,0	4,4	5,3	6,4	7,1	8,0	1,8	6,2	1,3
Treligorebi	13	-20,4	-18,3	-16,8	-15,4	-13,7	2,9	-16,8	2,1	13,0	4,4	5,3	6,4	7,1	8,0	1,8	6,2	1,3

**Middle Bronze Age/area**	δ13C (‰)									δ15N (‰)								
	n	min	Q1	med	Q3	max	IQR	mean	sd	n	min	Q1	med	Q3	max	IQR	mean	sd
Middle Bronze Age	77	-21,2	-19,2	-18,3	-17,7	-15,2	1,5	-18,3	1,3	77,0	2,1	4,8	6,0	6,7	8,5	1,9	5,8	1,3
Samtskhe-Javakheti	31	-20,0	-19,4	-18,5	-18,0	-16,6	1,4	-18,6	0,9	31,0	2,1	4,1	4,8	6,2	8,1	2,1	5,2	1,6
Atskuri	31	-20,0	-19,4	-18,5	-18,0	-16,6	1,4	-18,6	0,9	31,0	2,1	4,1	4,8	6,2	8,1	2,1	5,2	1,6
Kvemo Kartli	13	-20,0	-18,9	-18,2	-16,6	-15,5	2,3	-17,8	1,5	13,0	5,8	6,2	6,7	7,0	8,1	0,8	6,7	0,7
Gantiadi	4	-19,4	--	--	--	-15,5	--	--	--	4,0	6,2	--	--	--	7,1	0,3	--	--
Karataki	9	-20,0	-18,8	-17,6	-16,6	-15,8	2,2	--	--	9,0	5,8	6,1	6,2	7,0	8,1	0,9	--	--
Mtskheta-Mtianeti	31	-21,2	-19,0	-18,1	-17,3	-15,2	1,8	-18,0	1,5	31,0	3,9	5,4	6,0	6,5	8,5	1,0	6,0	1,0
Abanoskhevi	8	-21,2	-19,4	-19,1	-18,3	-18,1	1,1	--	--	8,0	4,2	5,3	5,5	5,9	6,5	0,6	--	--
Natakhtari	7	-18,4	-18,1	-17,9	-17,6	-16,4	0,6	--	--	7,0	5,7	6,6	6,8	7,3	8,5	0,7	--	--
Samtavro	6	-20,4	-19,3	-18,9	-18,7	-18,0	0,6	--	--	6,0	3,9	5,7	6,1	6,8	7,6	1,1	--	--
Tsitsamuri	10	-19,2	-17,7	-16,4	-15,6	-15,2	2,1	-16,8	1,5	10,0	4,4	5,3	5,8	6,1	6,2	0,8	5,6	0,6
Shida Kartli	2	-19,3	--	--	--	-18,8	--	--	--	2,0	5,8	--	--	--	7,9	--	--	--
Tsaghvli	2	-19,3	--	--	--	-18,8	--	--	--	2,0	5,8	--	--	--	7,9	--	--	--

**Late Bronze Age/area**	δ13C (‰)									δ15N (‰)								
	n	min	Q1	med	Q3	max	IQR	mean	sd	n	min	Q1	med	Q3	max	IQR	mean	sd
Late Bronze Age	33	-20,9	-19,7	-18,3	-16,2	-13,7	3,5	-17,8	2,2	33,0	4,0	5,2	5,5	6,7	8,0	1,5	5,8	1,1
Mtskheta-Mtianeti	17	-20,9	-19,7	-18,5	-17,1	-13,9	2,6	-18,1	2,0	17,0	4,0	5,2	5,3	6,0	7,7	0,8	5,5	0,9
Abanoskhevi	9	-20,9	-19,4	-18,5	-17,8	-16,8	1,6	--	--	9,0	5,2	5,2	5,5	6,0	7,7	0,8	--	--
Samtavro	8	-20,1	-19,7	-18,4	-15,4	-13,9	4,3	--	--	8,0	4,0	4,5	4,8	5,7	7,0	1,2	--	--
North Caucasus	3	-20,8	--	--	--	-20,3	--	--	--	3,0	4,1	--	--	--	6,8	--	--	--
Koban	3	-20,8	--	--	--	-20,3	--	--	--	3,0	4,1	--	--	--	6,8	--	--	--
Tbilisi district	13	-20,4	-18,3	-16,8	-15,4	-13,7	2,9	-16,8	2,1	13,0	4,4	5,3	6,4	7,1	8,0	1,8	6,2	1,3
Treligorebi	13	-20,4	-18,3	-16,8	-15,4	-13,7	2,9	-16,8	2,1	13,0	4,4	5,3	6,4	7,1	8,0	1,8	6,2	1,3

min: minimum; med: median; max: maximum; ; sd: standard deviation

Q1: first quartile (the lowest 25% of numbers); Q3: third quartile (the 51% to 75% above the median); IQR: interquartile range

SI appendix

Table S4.3

Descriptive statistics of animal carbon and nitrogen isotopic ratios according to animal groups (bovid, caprid), chronological periods and geographical areas

	$\delta^{13}C$ (‰)									$\delta^{15}N$ (‰)								
	n	min	Q1	med	Q3	max	IQR	mean	sd	n	min	Q1	med	Q3	max	IQR	mean	sd
**Animals**	**110**	**-21,2**	**-19,4**	**-18,3**	**-17,3**	**-13,7**	**2,1**	**-18,1**	**1,6**	**110**	**2,1**	**5,0**	**5,9**	**6,7**	**8,5**	**1,8**	**5,8**	**1,3**

**Middle Bronze Age/species**	$\delta^{13}C$ (‰)									$\delta^{15}N$ (‰)								
	n	min	Q1	med	Q3	max	IQR	mean	sd	n	min	Q1	med	Q3	max	mean	sd	IQR
Bovid	27	3,4	4,8	5,6	6,2	7,6	1,4	5,5	1,1	27	-20,0	-18,5	-17,9	-16,7	-15,2	-17,6	1,3	1,8
Canid	1	8,1	--	--	--	--	--	--	--	1	-15,8	--	--	--	--	--	--	--
Caprid	34	2,1	4,7	6,0	6,9	8,5	2,2	5,7	1,5	34	-20,0	-19,3	-18,8	-18,1	-15,5	-18,6	1,0	1,3
Equid	1	4,6	--	--	--	--	--	--	--	1	-18,2	--	--	--	--	--	--	--
Fowl	1	8,1	--	--	--	--	--	--	--	1	-17,9	--	--	--	--	--	--	--
Small game	1	6,0	--	--	--	--	--	--	--	1	-20,0	--	--	--	--	--	--	--
Suid	10	4,8	6,1	6,4	7,1	8,0	1,0	6,5	1,0	10	-21,2	-19,4	-19,0	-18,1	-17,8	-19,0	1,1	1,4
Wild herbivore	2	3,9	--	--	--	6,7	--	--	--	2	-20,4	--	--	--	-15,5	--	--	--

**Late Bronze Age/species**	$\delta^{13}C$ (‰)									$\delta^{15}N$ (‰)								
	n	min	Q1	med	Q3	max	IQR	mean	sd	n	min	Q1	med	Q3	max	mean	sd	IQR
Bovid	12	4,4	4,5	5,2	5,7	8,0	1,1	5,4	1,1	12	-19,9	-18,3	-17,5	-15,6	-13,8	-17,1	1,9	2,7
Caprid	16	4,0	5,3	5,8	6,5	8,0	1,2	5,9	1,2	16	-20,8	-19,8	-18,9	-16,8	-13,7	-18,1	2,3	3,0
Equid	2	5,2	--	--	--	5,9	--	--	--	2	-20,9	--	--	--	-18,5	--	--	--
Suid	2	6,7	--	--	--	6,8	--	--	--	2	-20,4	--	--	--	-17,8	--	--	--
Wild herbivore	1	7,7	--	--	--	--	--	--	--	1	-14,7	--	--	--	--	--	--	--

**Bovid**	$\delta^{13}C$ (‰)									$\delta^{15}N$ (‰)								
	n	min	Q1	med	Q3	max	IQR	mean	sd	n	min	Q1	med	Q3	max	IQR	mean	sd
LBA	12	4,4	4,5	5,2	5,7	8,0	1,1	5,4	1,1	12	-19,9	-18,3	-17,5	-15,6	-13,8	-17,1	1,9	2,7
MBA	27	3,4	4,8	5,6	6,2	7,6	1,4	5,5	1,1	27	-20,0	-18,5	-17,9	-16,7	-15,2	-17,6	1,3	1,8
Samtskhe-Javakheti	10	3,4	4,2	4,5	5,0	6,7	0,9	4,6	0,9	10	-20,0	-19,3	-18,4	-17,6	-16,6	-18,4	1,2	1,7
Atskuri	10	3,4	4,2	4,5	5,0	6,7	0,9	4,6	0,9	10	-20,0	-19,3	-18,4	-17,6	-16,6	-18,4	1,2	1,7
Kvemo Kartli	4	6,0	--	--	--	7,6	--	--	--	4	-18,2	--	--	--	-16,1	--	--	--
Gantiadi	1	6,2	--	--	--	--	--	--	--	1	-18,2	--	--	--	--	--	--	--
Karataki	3	6,0	--	--	--	7,6	--	--	--	3	-16,9	--	--	--	-16,1	--	--	--
Mtskheta-Mtianeti	19	4,4	5,2	5,6	6,2	7,6	1,0	5,6	0,8	19	-19,9	-18,3	-17,8	-15,9	-14,7	-17,3	1,5	2,4
Abanoskhevi	5	5,2	--	--	--	6,2	--	--	--	5	-19,9	--	--	--	-17,8	--	--	--
Natakhtari	1	6,8	--	--	--	--	--	--	--	1	-18,4	--	--	--	--	--	--	--
Samtavro	6	4,4	4,7	5,4	6,0	7,6	1,4	--	--	6	-18,8	-18,5	-17,6	-16,0	-14,7	-17,2	--	--
Tsitsamuri	7	5,0	5,4	5,7	6,1	6,2	0,7	--	--	7	-17,9	-16,9	-15,9	-15,6	-15,2	-16,3	--	--
Tbilisi district	6	4,4	4,7	5,2	6,7	8,0	2,0	--	--	6	-19,4	-18,3	-17,2	-15,6	-13,8	-16,9	--	--
Treligorebi	6	4,4	4,7	5,2	6,7	8,0	2,0	--	--	6	-19,4	-18,3	-17,2	-15,6	-13,8	-16,9	--	--

**Caprid**	$\delta^{13}C$ (‰)									$\delta^{15}N$ (‰)								
	n	min	Q1	med	Q3	max	IQR	mean	sd	n	min	Q1	med	Q3	max	mean	sd	IQR
LBA	16	4,0	5,3	5,8	6,5	8,0	1,2	5,9	1,2	16	-20,8	-19,8	-18,9	-16,8	-13,7	-18,1	2,3	3,0
MBA	34	2,1	4,7	6,0	6,9	8,5	2,2	5,7	1,5	34	-20,0	-19,3	-18,8	-18,1	-15,5	-18,6	1,0	1,3
Samtskhe-Javakheti	9	2,1	3,4	3,7	4,7	7,4	1,3	--	--	9	-19,8	-19,1	-18,7	-18,1	-17,6	-18,7	--	--
Atskuri	9	2,1	3,4	3,7	4,7	7,4	1,3	--	--	9	-19,8	-19,1	-18,7	-18,1	-17,6	-18,7	--	--
Kvemo Kartli	7	5,8	6,2	6,7	7,0	7,1	0,8	--	--	7	-20,0	-19,4	-18,9	-18,6	-17,6	-18,9	--	--
Gantiadi	2	6,7	--	--	--	7,1	0,2	--	--	2	-19,4	--	--	--	-18,9	-19,1	--	--
Karataki	5	5,8	--	--	--	7,0	0,8	--	--	5	-20,0	--	--	--	-17,6	-18,8	--	--
Mtskheta-Mtianeti	25	4,0	5,4	6,0	6,7	8,5	1,3	6,0	1,1	25	-20,1	-19,4	-18,8	-17,7	-13,9	-18,3	1,5	1,7
Abanoskhevi	9	4,2	5,3	5,5	6,0	7,7	0,7	--	--	9	-19,4	-19,4	-18,8	-18,1	-16,8	-18,5	--	--
Natakhtari	6	5,7	6,5	6,9	7,5	8,5	1,0	--	--	6	-18,2	-18,0	-17,8	-17,5	-16,4	-17,6	--	--
Samtavro	7	4,0	5,0	5,9	6,6	7,0	1,6	--	--	7	-20,1	-19,7	-19,7	-19,2	-13,9	-18,8	--	--
Tsitsamuri	3	4,4	--	--	--	6,2	0,9	--	--	3	-19,2	--	--	--	-15,5	--	--	--
North Caucasus	2	4,1	--	--	--	6,5	1,2	--	--	2	-20,8	--	--	--	-20,3	--	--	--
Koban	2	4,1	--	--	--	6,5	1,2	--	--	2	-20,8	--	--	--	-20,3	--	--	--
Shida Kartli	2	5,8	--	--	--	7,9	1,0	--	--	2	-19,3	--	--	--	-18,8	--	--	--
Tsaghvli	2	5,8	--	--	--	7,9	1,0	--	--	2	-19,3	--	--	--	-18,8	--	--	--
Tbilisi district	5	5,3	--	--	--	8,0	1,3	--	--	5	-20,4	--	--	--	-13,7	--	--	--
Treligorebi	5	5,3	--	--	--	8,0	1,3	--	--	5	-20,4	--	--	--	-13,7	--	--	--

**Suid**	$\delta^{13}C$ (‰)									$\delta^{15}N$ (‰)								
	n	min	Q1	med	Q3	max	IQR	mean	sd	n	min	Q1	med	Q3	max	mean	sd	IQR
LBA	2	6,7	--	--	--	6,8	--	--	--	2	-20,4	--	--	--	-17,8	--	--	--
MBA	10	4,8	6,1	6,4	7,1	8,0	1,0	6,5	1,0	10	-21,2	-19,4	-19,0	-18,1	-17,8	-19,0	1,1	1,4
Samtskhe-Javakheti	9	4,8	6,1	6,2	7,2	8,0	1,1	--	--	9	-20,0	-19,3	-19,0	-18,0	-17,8	-18,7	--	--
Atskuri	9	4,8	6,1	6,2	7,2	8,0	1,1	--	--	9	-20,0	-19,3	-19,0	-18,0	-17,8	-18,7	--	--
Mtskheta-Mtianeti	1	6,5	--	--	--	--	--	--	--	1	-21,2	--	--	--	--	--	--	--
Abanoskhevi	1	6,5	--	--	--	--	--	--	--	1	-21,2	--	--	--	--	--	--	--
North Caucasus	1	6,8	--	--	--	--	--	--	--	1	-20,4	--	--	--	--	--	--	--
Koban	1	6,8	--	--	--	--	--	--	--	1	-20,4	--	--	--	--	--	--	--
Tbilisi district	1	6,7	--	--	--	--	--	--	--	1	-17,8	--	--	--	--	--	--	--
Treligorebi	1	6,7	--	--	--	--	--	--	--	1	-17,8	--	--	--	--	--	--	--

min: minimum; med: median; max: maximum; ; sd: standard deviation

Q1: first quartile (the lowest 25% of numbers); Q3: third quartile (the 51% to 75% above the median); IQR: interquartile range

SI Appendix table S5 Results of statistical tests performed on human and animal carbon isotope ratios using R©3.6.1

Statistical analyses were performed using the statistical program R (version 3.6.1) and RStudio (version 1.2.5033) (R Development Core Team, 2005; R Core Team, 2018). The non-parametric tests, Mann-Whitney-Wilcoxon and Kruskal-Wallis with FDR correction, were used to compare between series.

R Development Core Team. 2005. A language and environment for statistical computing. R Foundation for Statistical Computing. Vienna, Austria: ISBN 3-900051-07-0, URL: http://www.R-project.org.
R Core Team. 2018. R vesion 3.6.1 (R foundation for Statistical Computing). Vienna, Austria.

```
> pairwise.wilcox.test(Human$d13C, Human$Chrono, p.adjust.method = "fdr")
Pairwise comparisons using Wilcoxon rank sum test with continuity correction
data:  Human$d13C and Human$Chrono
              G-LBA
G-MBA         1.5e-12
P value adjustment method: fdr
Warning message:
In wilcox.test.default(xi, xj, paired = paired, ...) :
  impossible de calculer la p-value exacte avec des ex-aequos
```

```
> pairwise.wilcox.test(Human$d15N, Human$Chrono, p.adjust.method = "fdr")
Pairwise comparisons using Wilcoxon rank sum test with continuity correction
data:  Human$d15N and Human$Chrono
          G-LBA
G-MBA     1.9e-05
P value adjustment method: fdr
Warning message:
In wilcox.test.default(xi, xj, paired = paired, ...) :
  impossible de calculer la p-value exacte avec des ex-aequos
```

```
> pairwise.wilcox.test(Fauna$d13C, Fauna$Chrono, p.adjust.method = "fdr")
Pairwise comparisons using Wilcoxon rank sum test with continuity correction
data:  Fauna$d13C and Fauna$Chrono
              G-LBA
G-MBA          0.59
P value adjustment method: fdr
```

```
> pairwise.wilcox.test(Fauna$d15N, Fauna$Chrono, p.adjust.method = "fdr")
Pairwise comparisons using Wilcoxon rank sum test with continuity correction
data:  Fauna$d15N and Fauna$Chrono
          G-LBA
G-MBA     0.81
P value adjustment method: fdr
```

```
> pairwise.wilcox.test(Bovid$d13C, Bovid$Chrono, p.adjust.method = "fdr")
Pairwise comparisons using Wilcoxon rank sum test with continuity correction
data:  Bovid$d13C and Bovid$Chrono
              G-LBA
G-MBA          0.44
P value adjustment method: fdr
Warning message:
In wilcox.test.default(xi, xj, paired = paired, ...) :
  impossible de calculer la p-value exacte avec des ex-aequos
```

```
> pairwise.wilcox.test(Bovid$d15N, Bovid$Chrono, p.adjust.method = "fdr")
Pairwise comparisons using Wilcoxon rank sum test with continuity correction
data:  Bovid$d15N and Bovid$Chrono
          G-LBA
G-MBA     0.54
P value adjustment method: fdr
Warning message:
In wilcox.test.default(xi, xj, paired = paired, ...) :
  impossible de calculer la p-value exacte avec des ex-aequos
```

```
> pairwise.wilcox.test(Caprid$d13C, Caprid$Chrono, p.adjust.method = "fdr")
Pairwise comparisons using Wilcoxon rank sum test with continuity correction
data:  Caprid$d13C and Caprid$Chrono
              G-LBA
G-MBA          0.82
P value adjustment method: fdr
Warning message:
In wilcox.test.default(xi, xj, paired = paired, ...) :
  impossible de calculer la p-value exacte avec des ex-aequos
```

```
> pairwise.wilcox.test(Caprid$d15N, Caprid$Chrono, p.adjust.method = "fdr")
Pairwise comparisons using Wilcoxon rank sum test with continuity correction
data:  Caprid$d15N and Caprid$Chrono
          G-LBA
G-MBA     0.94
P value adjustment method: fdr
Warning message:
In wilcox.test.default(xi, xj, paired = paired, ...) :
  impossible de calculer la p-value exacte avec des ex-aequos
```

SI appendix figure S3

Box-plots of human carbon isotope ratios

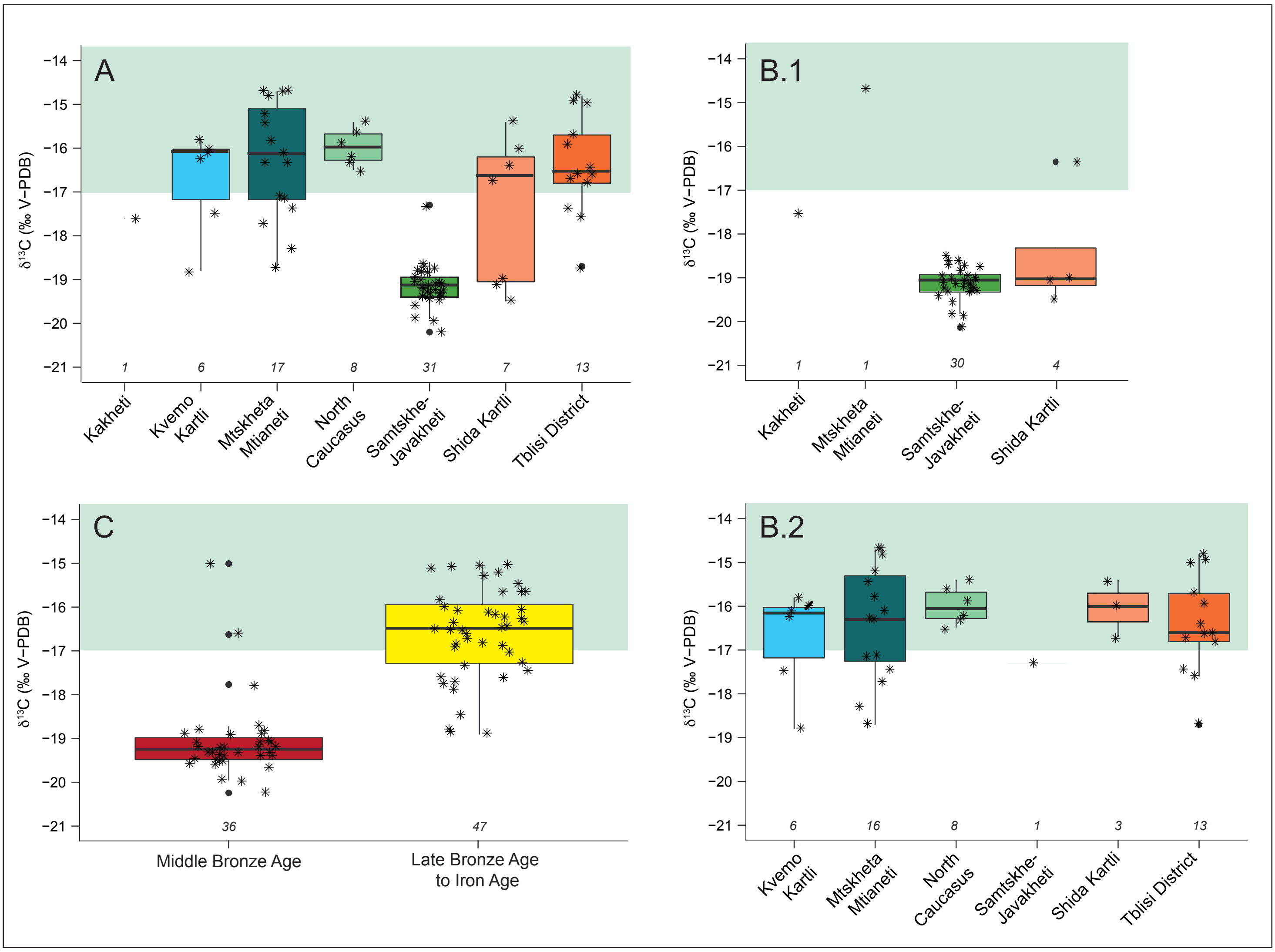

Figure S3.1. Box-plots of human carbon isotope ratios according to geographical areas (A), to chronological groups and geographical areas (B1: Middle Bronze Age, B2: Late Bronze Age to Late Iron Age), and human pooled together according to chronological groups (C)

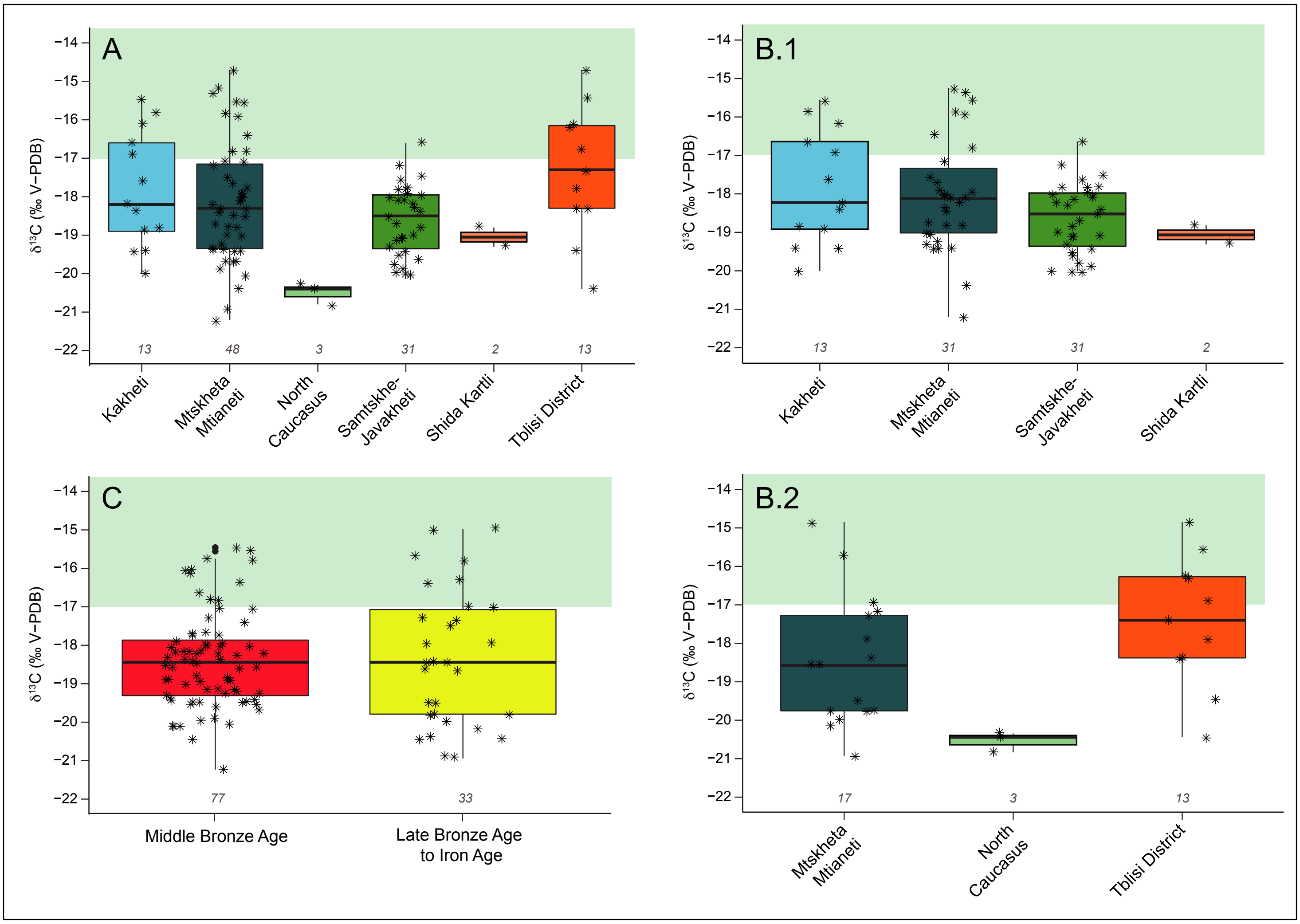

Figure S3.2: Box-plots of animal carbon isotope ratios according to geographical areas (A), to chronological groups and geographical areas (B1: Middle Bronze Age, B2: Late Bronze Age to Late Iron Age), and human pooled together according to chronological groups (C).

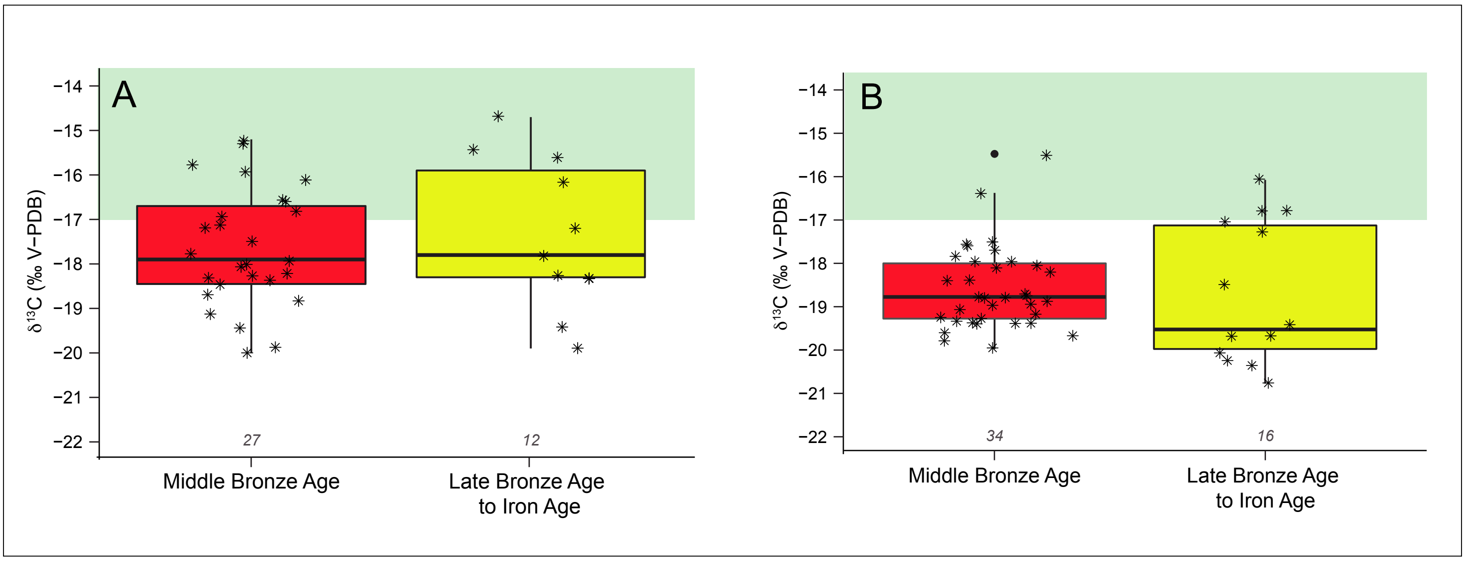

Figure S3.3. Box-plots of animal carbon isotope ratios according to chronological groups for bovid (A) and caprid (B).

Supplementary information references

Table S1

1. E. Lebedeva, "The first results of archaeobotanical studies at the archaeological sites of Adygea" in Analytical researches of Laboratory of Nature sciences in archaeology, vol. 2 (Moscow, 2011), pp. 244-257, in Russian.
2. V. A. Trifonov, N. I. Shishlina, E. Y. Lebedeva, J. van der Plicht, S. A. Rishko, Directly dated broomcorn millet from the northwestern Caucasus: Tracing the Late Bronze Age route into the Russian steppe. *Journal of Archaeological Science: Reports* **12**, 288-294 (2017).
3. E. Chantre, *Recherches anthropologiques dans le Caucase. Tome 2 : Période Protohistorique* (Paris, ed. Reinwald, 1886).
4. C. Knipper, S. Reinhold, J. Gresky, A. Belinskiy, K. W. Alt, "Economic strategies at Bronze Age and Early Iron Age upland sites in the North Caucasus: Archaeological and stable isotope investigations" in Isotopic Investigations of Pastoral Production: Innovative Approaches to Patterns of Mobility, Economy, and Exploitation, A. R. Vantresca Miller, C. A. Makarewicz, Eds. (2018), pp. 123-140.
5. G. Bedianashvili, C. Bodet, Koban necropolis, tombs 9 and 12: The Late Bronze to the Early Iron Age of the Northern Caucasus. *TÜBA-AR* **13**, 276-294 (2010).
6. E. Herrscher *et al.*, The origins of millet cultivation in the Caucasus: archaeological and archaeometric approaches. *Préhistoires Méditerranéennes* **6** (2018).
7. E. Lebedeva, "On the mountain agriculture in Ossetia in the Bronze Age (archaeobotanical studies at Chidgom settlement)" in Materials of protecting archaeological researches: towns, settlements, cemeteries, vol. 17. (Cheboksary, 2015), pp. 68-77, in Russian.
8. N. I. Shishlina *et al.*, Plant food subsistence in the human diet of the Bronze Age Caspian and Low Don steppe pastoralists: archaeobotanical, isotope and 14C data. *Vegetation History and Archaeobotany* **27**, 833-842 (2018).
9. P. L. Kohl, R. G. Magomedov, Bronze Developments on the West Caspian Coastal Plain. *Paleorient* **40**, 93-114 (2014).
10. G. N. Lisitsina, L. V. Prishchepenko, "Paleoetnobotanicheskie nachodki Kavkaza i Blijnego Vostoka [Palaeoethnobotanical Finds of the Caucasus and the near East]". (Moscow, 1977), pp. 5-125, in Russian.
11. N. Rusishvili, *Cultivated Plants in ancient territory of Georgia by paleoethnobotanical research*, Academy of Science of Moldovan SSR (PhD Thesis, University of Moldova, Botanical Garden, Chișinău, 1990) in Russian.
12. A. Sagona, *The Archaeology of the Caucasus. From Earliest Settlements to the Iron Age*, Cambridge World Archaeology (ed. Cambridge University Press, 2018).
13. O. Lordkipanidze, Vani: An Ancient City of Colchis. *Greek, Roman, and Byzantine Studies* **32**, 151-195 (1991).
14. I. Chavleishvili, *Late Bronze-Early Iron Age Settlements from South-West Georgia.*, PHD thesis (Tbilisi, 1999).
15. A. Ramishvili, *Arkheologicheskie raboti v c. Tsagvli/Archaeological (works in village Tsagvli) – Polevie Arkhelogicheskie Isledovanya v. 1979 (Field Archaeological Investigation [Short Reports] in 1979)* (1982), pp. 20-21, in Georgian.
16. A. Ramishvili, *Raskopki v natsargora (Khashurki raion) (Excavations at Natsargora (Khashuri district) – Polevie Arkheologicheskiye issledovaniya v. 1988 (Field Archaeological Investigation [Short Reports] in 1988)* (1997), pp. 40-44. in Russian.
17. A. Ramishvili, *Shida Kartlis Brinjaos khanis finaluri stadiebis arqeologiuri problemebi (Archaeological problems of final phases of Bronze Age in Shida Kartli)*, Centre for Archaeological Studies (PhD Thesis, Tbilisi, 1998) in Georgian.
18. G. Mindiashvili, S. Iremishvili, Z. Sherazadishvili, Gudabertka Settlement (excavations of 2005, 2009 years). *Studies in Caucasian Archaeology* **I**, 234-250 (2012).

19. V. Licheli, Urban Development in Central Transcaucasia in Anatolian Context: New Data. *Ancient Civilizations from Scythia to Siberia* **17**, 135-156 (2011).

20. G. Gogochuri, "Archaeological sites of the Early Barrow Period in the Aragvi Gorge" in Archaeology in Southern Caucasus: Perspectives from Georgia*,* A. Sagona, Ed. (Ancient Near Eastern Studies, Supplement Series 19, 2008), pp. 37-62.

21. A. Kalandzadze *et al.*, "Short report on the archaeological investigation in the Greater Mtskheta territory" in Palyvie Archeologicheskie Isledovanie. (Metsniereba, 1979), pp. 149-165.

22. A. Apakidze *et al.*, *Arkheologicheskie Raskopki v Mtskheta y ee okruge. (Archaeological excavations in Mtskheta and its adjacent area)* (Metsniereba, Tbilisi, 1987), in Russian.

23. A. Apakidze, V. Nikolaishvili, G. Giunashvili, R. Davlianidze, G. Narimanishvili, *The Mtskheta Expedition, Field-work investigations in 1990-1992 (Short reports)* (Metsniereba, Tbilisi, 2004), in Georgian.

24. V. Sadradze, *Mtskhetisa da misi shemogarenis dzv.ts. II-I atastsleulis pirveli nakhevris arkeologiuri dzegleb (Archaeological Sites in Mtskheta and its Environs of the First half of the II-I Millennia BC)* (Tbilisi, 2002), in Georgian.

25. T. Chubinishvili, *Mtskhetis udzvelesi archeologiuri dzeglebi (Ancient archaeological sites of Mtskheta)* (Teqnika da shroma, Tbilisi, 1957), in Georgian.

26. V. Nikolaishvili, E. Gavasheli, *Narekvavis arqeologiuri dzeglebi (Narekvavi archaeological sites)* (2007), in Georgian.

27. R. M. Abramishvili, "Archäologische Denkmäler in Tbilisi" in Unterwegs zum Goldenen Vlies. Archäologische Funde aus Georgien*,* A. Miron, W. Orthmann, Eds. (Theiss, Stuttgart, Saarbrücke, 1995), pp. 187-196.

28. V. Licheli *et al.*, *Archaeological Investigation at Site IV- 266/320, KP211/212, Atskuri Village, Akhaltsikhe Region* (Otar Lordkipanidze Centre of Archaeology of the Georgian National Museum, Tbilisi, 2007).

29. V. Licheli, A burial with a stone embankment at Atsquri. *Journal of Georgian Archaeeology* **1**, 218-223 (2004).

30. G. Gogochuri, A. Orjonikidze, *Comprehensive Technical Report on Archaeological Investigations at site IV-293 Tiselis Seri KP 203*, unpublished report submitted to BTC and SCP Pipeline Companies (Tbilisi, 2007).

31. O. Japaridze, I. Kikvidze, G. Avalishvili, A. Tsereteli, *The results of Archaeological Excavations in Meskhet-Javakheti* (Metsniereba, Tbilisi, 1981).

32. N. Tushishvili, *The results of archaeological investigations in Algeti gorge – Polevie Arkheologicheskiye issledovaniya v. 1979 (Field Archaeological Investigation [Short Reports] in 1979)* (Metsniereba, Tbilisi, 1982), pp. 47-56, in Russian.

33. N. Tushishvili, J. Amiranashvili, *Arxeologicheskie raskopki v zone stroitelstva Algetskogo vodoxranilishcha Archaeological Excavations in the Area of the Algeti Storage Reservoir – Arxeologicheskie isledovaniia na novostroikax Gruzinskoi SSR (Archaeological Studies at New Construction Sites of Georgian SSR)* (Tbilisi, 1982), pp. 65-74, in Russian.

34. K. Kakhiani, M. Gvligashvili, V. Sadradze, G. Kalandadze, Z. Tskvitinidze, *Archaeological Investigations of Mashavera gorge – Polevie Arkheologicheskiye issledovaniya v. 1984-86 (Field Archaeological Investigation [Short Reports] in 1984-86)* (Metsniereba, Tbilisi, 1991), pp. 51-59, in Russian.

35. K. Kakhiani, E. Gligvashvili, "Bronze age barrows in the southeast Georgia" in Archaeology in Southern Caucasus: Perspectives from Georgia*,* A. Sagona, Ed. (Ancient Near Eastern Studies, Supplement Series 19, 2008), pp. 229-247.

36. K. Kakhiani *et al.*, "Archaeological excavations in Dmanisi district in 1989-1990. Archaeological field investigations in 1989-1992". (Tbilisi, 2004), pp. 39-44.

37. S. Hansen, G. Mirtskhulava, K. Bastert-Lamprichs, Aruchlo: A Neolithic Settlement Mound in the Caucasus (field report). *Neo-Lithics* **1**, 13-19 (2007).

38. C. Hamon, From Neolithic to Chalcolithic in the Southern Caucasus: Economy and Macrolithic Implements from Shulaveri-Shomu Sites of Kwemo-Kartli (Georgia). *Paléorient* **34**, 85-135 (2008).

39. K. Pitskhelauri, "Eastern Georgia at the end of the Bronze Age" in Proceeding of the Kakheti Archaeological expedition. Vol. III. (Tbilisi, 1979), in Russian.

40. A. Furtwängler, F. Knauss, Archäologische Expedition in Kachetien 1996. Ausgrabungen in den Siedlungen Gumbati und Ciskaraant Gora. *Eurasia Antiqua* **3**, 353-387 (1997).

41. A. Furtwängler, F. Knauss, Archäologische Expedition in Kachetien. Ausgrabungen in Siraki. *Eurasia Antiqua* **5**, 233-270 (1998).

42. A. Furtwängler, F. Knauss, I. Motzenbäcker, Archäologische Expedition in Kachetien. Ausgrabungen in Siraki. *Eurasia Antiqua* **4**, 309-364 (1998).

43. R. Hovsepyan, Archaeobotanical Findings from Yenokavan-2 Cave Site (Armenia). *Biological Journal of Armenia* **63**, 83-84 (2011).

44. R. Badalyan, A. T. Smith, I. Lindsay, L. Khatchadourian, P. S. Avetisyan, Village, fortress, and town in Bronze and Iron Age Southern Caucasia: A preliminary report on the 2003-2006 investigations of Project ArAGATS on the Tsaghkahovit Plain, Republic of Armenia. *Archäologische Mitteilungenaus Iran und Turan* **40**, 96-105 (2008).

45. R. Badalyan *et al.*, A Preliminary Report on the 2008, 2010, and 2011 Investigations of Project ArAGATS on the Tsaghkahovit Plain, Republic of Armenia. *Archäologische Mitteilungenaus Iran und Turan* **46**, 149-222 (2014).

46. R. Hovsepyan, "Palaeoethnobotanical material from pots of tomb N3 of Nerkin Naver tombfield" in Archaeology, Ethnography and Folklore of the Caucasus. (Epokha, Makhachkala, 2007), pp. 215-217, in Russian.

47. L. Khatchadourian, "Unforgettable Landscapes: Attachments to the past in Hellenistic Armenia" in Negotiating the Past in the Past: Identity, Memory, and Landscape in Archaeological Research, N. Yoffee, Ed. (University of Arizona Press, Tucson, 2007), pp. 58-59.

48. B. Piotrovskii, *Karmir-Blur 3: Resultat Reskopok 1951–1953* (Akademiya Nauk Armianskoi SSSR, Yerevan, 1955), in Russian.

49. H. Avetisyan, W. Allinger-Csollich, The Fortress of Aramus: Preliminary Report of Excavations in 2004 and 2005. *Aramazd. Armenian Journal of Near Eastern Studies (AJNES)* **I**, 105-134 (2006).

50. W. Kuntner, S. Heinsch (2010) The Ostburg of Aramus, an urartian and achaemenid fortress. The stratigraphical evidence. in *Vol. 2: Excavations, Surveys and Restorations: Reports on Recent Field Archaeology in the Near East. Proceedings of the 6th International Congress on the Archaeology of the Ancient Near East.*, eds P. Matthiae, F. Pinnock, L. Nigro, N. Marchetti (Wiesbaden, Harrassowitz Verlag), pp. 339-348.

51. K. K. Kushnareva, Poselenie epokhi bronzy na kholme Uzerlik-tepe, okolo Agdama. *Materialy i issledovaniia po arkheologii SSSR* **67** (Moscow-Leningrad,1959), in Russian.

52. K. K. Kushnareva, Novye dannye o poselenii Uzerlik-tepe, okolo Agdama *Materialy i issledovaniia po arkheologii SSSR* **125**, (Moscow-Leningrad,1965), in Russian.

53. C. Longford, A. Drinnan, A. Sagona, "Archaeobotany of Sos Höyük, northeast Turkey" in New Directions in Archaeological Science, vol. 28, A. Fairbairn, S. O'Connor, B. Marwick, Eds. (Canberra, 2009), pp. 121-136.

54. T. Solmaz, E. Oybak Dönmez, Archaeobotanical studies at the Urartian site of Ayanis in Van Province, eastern Turkey. *Turkish Journal of Botany* **37**, 282-296 (2013).

55. M. Nesbitt, G. D. Summers, Some recent discoveries of millet (*Panicum miliaceum* L. and *Setaria italica* (L.) P. BEAUV.) at excavations in Turkey and Iran. *Anatolian Studies* **38**, 85-97 (1988).

56. C. Bronk Ramsey, Methods for Summarizing Radiocarbon Datasets. *Radiocarbon* **59**, 1809-1833 (2017).

57. P. J. Reimer *et al.*, IntCal13 and Marine13 Radiocarbon Age Calibration Curves 0–50,000 Years cal BP. *Radiocarbon* **55**, 1869-1887 (2013).
