# Supplementary material for: The place of millet in food globalization during Late Prehistory as evidenced by new bioarchaeological data from the Caucasus
Source: Sci Rep. 2021 Jun 23;11:13124. doi: 10.1038/s41598-021-92392-9 (PMC8222238; doi:10.1038/s41598-021-92392-9)
Supplement: Supplementary file 2 — Supplementary Information 2. [file 41598_2021_92392_MOESM2_ESM.pdf]

DATASET S1

MARTIN et al The place of millet in food globalization during Late Prehistory as evidenced by new bioarchaeological data from the Caucasus

Poz-82832-Velikent I R_Date(1435±30)
68.2% probability
604AD (68.2%) 646AD
95.4% probability
570AD (95.4%) 655AD

Poz-82834-Velikent I R_Date(1490±30)
68.2% probability
549AD (68.2%) 605AD
95.4% probability
436AD (1.2%) 446AD
472AD (2.0%) 486AD
534AD (92.2%) 644AD

Poz-66775-Vani R_Date(1615±30)
68.2% probability
396AD (35.1%) 432AD
490AD (33.1%) 532AD
95.4% probability
386AD (95.4%) 538AD

Poz-51399-Atskuri-settl. ATS H03 R_Date(2265±30)
68.2% probability
392BC (39.5%) 357BC
283BC (21.9%) 256BC
246BC (6.8%) 236BC
95.4% probability
399BC (44.4%) 350BC
306BC (51.0%) 209BC

Bln-5636-Noname Gora R_Date(2439±30)
68.2% probability
731BC (16.7%) 691BC
660BC (3.6%) 650BC
544BC (47.9%) 428BC
95.4% probability
751BC (23.1%) 682BC
669BC (8.7%) 636BC
626BC (1.5%) 614BC
592BC (62.2%) 408BC

Poz-82837-Lesnoe R_Date(2455±30)
68.2% probability
749BC (26.8%) 684BC
667BC (10.5%) 640BC
588BC (2.9%) 578BC
566BC (28.0%) 484BC
95.4% probability
756BC (28.0%) 679BC
671BC (17.8%) 606BC
598BC (49.6%) 413BC

Bln-5544-Noname Gora R_Date(2479±28)
68.2% probability
756BC (11.3%) 727BC
718BC (4.1%) 706BC
694BC (5.7%) 680BC
670BC (24.7%) 606BC
597BC (22.4%) 541BC
95.4% probability
772BC (95.4%) 486BC

Poz-66776-Narekvavi R_Date(2560±30)
68.2% probability
800BC (61.7%) 756BC
679BC (4.3%) 671BC
604BC (2.3%) 598BC
95.4% probability
805BC (66.3%) 746BC
686BC (7.5%) 666BC
643BC (21.6%) 553BC

Poz-66774-Trelogorebi R_Date(2565±30)
68.2% probability
802BC (68.2%) 761BC
95.4% probability
806BC (72.4%) 748BC
685BC (6.4%) 666BC
641BC (13.0%) 587BC
581BC (3.6%) 556BC

GrA-69633-Grakliani Gora GRK-1 R_Date(2820±35)
68.2% probability
1011BC (68.2%) 922BC
95.4% probability
1108BC (0.6%) 1100BC
1088BC (94.8%) 896BC

Le-4235-Guamsky Grot R_Date(2670±120)
68.2% probability
1009BC (62.3%) 751BC
682BC (1.9%) 668BC
636BC (1.2%) 626BC
614BC (2.9%) 592BC
95.4% probability
1124BC (94.3%) 472BC
465BC (0.5%) 452BC
446BC (0.6%) 430BC

Lyon-4369-Koban KH1os R_Date(2735±32)
68.2% probability
906BC (68.2%) 836BC
95.4% probability
970BC (1.9%) 960BC
936BC (93.5%) 812BC

Poz-66771-Gudarbertka R_Date(2815±30)
68.2% probability
1002BC (68.2%) 928BC
95.4% probability
1052BC (95.4%) 898BC

GrA-65338-Guamsky Grot R_Date(2835±35)
68.2% probability
1041BC (1.2%) 1038BC
1031BC (67.0%) 929BC
95.4% probability
1110BC (95.4%) 908BC

Poz-56372-Treli 2008±TRE H07 R_Date(2845±35)
68.2% probability
1050BC (53.6%) 970BC
960BC (14.6%) 935BC
95.4% probability
1114BC (95.4%) 916BC

Poz-82835-Chidgom R_Date(2870±30)
68.2% probability
1110BC (6.7%) 1097BC
1092BC (61.5%) 1002BC
95.4% probability
1127BC (95.4%) 931BC

Poz-51404-Grakliani Gora GRAK H01 R_Date(2935±30)
68.2% probability
1209BC (63.5%) 1108BC
1099BC (4.7%) 1089BC
95.4% probability
1224BC (95.4%) 1028BC

Sac-51401-Natakhtari NAT H07 R_Date(2935±30)
68.2% probability
1209BC (63.5%) 1108BC
1099BC (4.7%) 1089BC
95.4% probability
1224BC (95.4%) 1028BC

Poz-66780-Sös Hüyük R_Date(2945±35)
68.2% probability
1218BC (67.2%) 1110BC
1096BC (1.0%) 1094BC
95.4% probability
1261BC (95.4%) 1038BC

Poz-66779-Tsaghkahovit R_Date(2945±30)
68.2% probability
1212BC (68.2%) 1114BC
95.4% probability
1258BC (1.5%) 1247BC
1233BC (93.9%) 1048BC

Poz-82837-Kabardinka 2 R_Date(2950±30)
68.2% probability
1214BC (68.2%) 1118BC
95.4% probability
1260BC (3.2%) 1241BC
1236BC (92.2%) 1051BC

Poz-79379-Natakhtari NAT H10 R_Date(2955±35)
68.2% probability
1223BC (68.2%) 1115BC
95.4% probability
1269BC (95.4%) 1047BC

Poz-63429-Sös Hüyük R_Date(2960±35)
68.2% probability
1227BC (68.2%) 1118BC
95.4% probability
1276BC (95.4%) 1051BC

Sac-51411-Natakhtari NAT H04 R_Date(2965±30)
68.2% probability
1222BC (68.2%) 1126BC
95.4% probability
1268BC (95.4%) 1056BC

Poz-63429-Gegharot R_Date(2995±30)
68.2% probability
1279BC (62.3%) 1192BC
1143BC (5.9%) 1132BC
95.4% probability
1376BC (4.2%) 1352BC
1302BC (91.2%) 1122BC

Poz-82836-Chishkho R_Date(3000±30)
68.2% probability
1282BC (64.6%) 1195BC
1141BC (3.6%) 1134BC
95.4% probability
1376BC (6.0%) 1348BC
1304BC (89.4%) 1126BC

Sac-51402-Natakhtari NAT H08 R_Date(3015±30)
68.2% probability
1370BC (4.5%) 1360BC
1296BC (63.7%) 1214BC
95.4% probability
1387BC (15.3%) 1339BC
1316BC (74.7%) 1190BC
1178BC (2.5%) 1161BC
1144BC (2.8%) 1130BC

Sac-43669-Abanoskhevi ABA F11 R_Date(3030±30)
68.2% probability
1374BC (12.7%) 1354BC
1302BC (55.5%) 1226BC
95.4% probability
1396BC (94.9%) 1195BC
1139BC (0.5%) 1134BC

Poz-66772-Choloki R_Date(3130±35)
68.2% probability
1443BC (50.3%) 1384BC
1340BC (17.9%) 1311BC
95.4% probability
1496BC (5.3%) 1472BC
1463BC (90.1%) 1296BC

Poz-79360-Treli G13 H39 R_Date(3130±30)
68.2% probability
1438BC (54.6%) 1388BC
1338BC (13.6%) 1320BC
95.4% probability
1494BC (3.3%) 1477BC
1458BC (66.3%) 1371BC
1359BC (25.8%) 1300BC

Poz-66777-Namcheduri R_Date(3150±35)
68.2% probability
1494BC (9.9%) 1478BC
1456BC (58.3%) 1396BC
95.4% probability
1501BC (84.1%) 1377BC
1344BC (11.3%) 1305BC

Poz-79398-Tsaghvli G13 H14 R_Date(3185±35)
68.2% probability
1497BC (29.7%) 1471BC
1464BC (38.5%) 1431BC
95.4% probability
1528BC (95.4%) 1400BC

Poz-79378-Natakhtari NAT H06 R_Date(3260±35)
68.2% probability
1608BC (20.0%) 1581BC
1562BC (48.2%) 1500BC
95.4% probability
1620BC (95.4%) 1450BC

Poz-79377-Atskuri Kurgan G13 H04 R_Date(3455±35)
68.2% probability
1874BC (18.0%) 1843BC
1816BC (8.3%) 1799BC
1779BC (29.0%) 1736BC
1716BC (13.0%) 1694BC
95.4% probability
1883BC (95.4%) 1687BC

Poz-79363-Natakhtari NAT F04 R_Date(3505±35)
68.2% probability
1886BC (15.2%) 1860BC
1853BC (53.0%) 1772BC
95.4% probability
1926BC (94.4%) 1742BC
1709BC (1.0%) 1701BC

Sac-51405-Karataki KAR F06 R_Date(3515±30)
68.2% probability
1890BC (15.6%) 1868BC
1848BC (52.6%) 1774BC
95.4% probability
1921BC (95.4%) 1751BC

Poz-79361-Tsistamuri TSI F10 R_Date(3520±35)
68.2% probability
1898BC (19.6%) 1866BC
1849BC (48.6%) 1774BC
95.4% probability
1939BC (95.4%) 1748BC

Sac-51407-Gantiadi GAN F03 R_Date(3530±30)
68.2% probability
1917BC (30.9%) 1872BC
1844BC (21.4%) 1812BC
1802BC (16.0%) 1777BC
95.4% probability
1942BC (95.4%) 1763BC

Sac-51403-Bertkana BER H10 R_Date(3530±30)
68.2% probability
1917BC (30.9%) 1872BC
1844BC (21.4%) 1812BC
1802BC (16.0%) 1777BC
95.4% probability
1942BC (95.4%) 1763BC

Poz-66778-Pichori R_Date(3550±35)
68.2% probability
1947BC (52.1%) 1876BC
1841BC (9.6%) 1821BC
1796BC (6.6%) 1782BC
95.4% probability
2010BC (1.6%) 2000BC
1977BC (93.8%) 1770BC

Sac-43672-Samtavro SAM F04 R_Date(3615±30)
68.2% probability
2022BC (68.2%) 1939BC
95.4% probability
2113BC (1.9%) 2100BC
2036BC (93.5%) 1892BC

Sac-43667-Abanoskhevi ABA F05 R_Date(3675±30)
68.2% probability
2133BC (38.2%) 2081BC
2060BC (25.9%) 2021BC
1992BC (4.1%) 1984BC
95.4% probability
2141BC (95.4%) 1956BC

Sac-43671-Gantiadi GAN F04 R_Date(3745±30)
68.2% probability
2202BC (56.3%) 2133BC
2081BC (11.9%) 2060BC
95.4% probability
2278BC (5.6%) 2250BC
2229BC (1.0%) 2221BC
2211BC (63.0%) 2112BC
2104BC (25.7%) 2036BC
